# Effectiveness of game-based learning of anesthetic drugs in anesthesiology residents: a pilot study

**DOI:** 10.1186/s12909-026-10011-5

**Published:** 2026-07-27

**Authors:** Santi Anchalee, Chanya Deekiatphaiboon, Sumidtra Prathep, Wilasinee Jitpakdee

**Affiliations:** Department of Anesthesiology, Faculty of Medicine, Prince of Songkla University, Hat Yai, Songkhla, 90110 Thailand

**Keywords:** Game-based learning, Card game, Anesthetic drug, Anesthesiology resident, Medical education

## Abstract

**Background:**

Active learning, particularly through serious and analog serious games, is effective for increasing participant engagement, fostering learners’ motivation, and promoting dynamic knowledge acquisition. In this study, we developed the “Basic Pharmacology for Anesthesia” card game to teach about anesthetic drug to first- to third-year anesthesiology residents and assessed its effectiveness and the residents’ satisfaction with game-based learning.

**Methods:**

Nineteen anesthesiology residents participated in this cross-sectional observational study conducted at the Department of Anesthesiology, Faculty of Medicine, Prince of Songkla University. A game-based learning was introduced to the participants. The objective of the game was for participants to collect a complete set of five cards for a specific anesthetic drug. The deck comprised 55 cards representing 11 anesthetic drugs, each of which detailed the drug’s name, dosage, onset and duration, contraindications, side effects, and metabolism or pharmacology. Pre- and posttests (total score: 20 points) were administered before and after the game-based learning, respectively. Pre- and posttest knowledge scores were compared, and the participants’ satisfaction with the activity was assessed using a five-point Likert scale.

**Results:**

A comparison of the pre- and posttest scores revealed improvements across all categories: 5.00 vs. 7.47 (*p* < 0.001) for induction drugs, 2.59 vs. 3.92 (*p* < 0.001) for volatile anesthetics, and 6.67 vs. 7.51 (*p* < 0.001) for neuromuscular agents. The total score significantly increased from 14.26 (pretest) to 18.91 (posttest) (*p* < 0.001). The overall satisfaction level was 4.84 (standard deviation = 0.37).

**Conclusions:**

Game-based learning using a serious card game could effectively improve anesthesiology residents’ knowledge of anesthetic drugs and was associated with a high learner satisfaction. To achieve a more comprehensive evaluation of this educational intervention, future studies should include additional assessment methods, such as measuring learner engagement and comparing game-based learning with traditional methods used to teach anesthetic drugs to anesthesiology residents.

**Supplementary Information:**

The online version contains supplementary material available at https://doi.org/10.1186/s12909-026-10011-5.

## Background

In the past, medical education predominantly relied on classical lectures and instructional materials, which lacked the flexibility to accommodate diverse student learning styles. This traditional approach often left students feeling bored and disengaged, struggling to retain information because of their limited interaction with instructors. As concerns grew over the effectiveness of the teacher-centered model, alternative teaching strategies gained prominence. Recent trends in educational research emphasize the importance of the learning process, innovative teaching methods, and curriculum development with the aim of fostering sustained educational success. For instance, active learning methods have been shown to enhance academic performance and student motivation while reducing learning challenges and failures [1].

Anesthesiologist training programs equip trainees with complex competencies necessary for their profession. Trainees must develop expertise in technical and non-technical skills to excel in this specialized field. A phenomenographic study [2] provided qualitative insights into such a learning process, categorizing the conceptions of anesthesiologists in learning about anesthesia into three themes: “learning competencies in anesthesia,” “learning the work of an anesthesiologist,” and “learning to become an anesthetic professional.” These themes are linked to six dimensions, one of which is *“*learning resources,*”* also referred to as fundamental theoretical knowledge. Within this framework, trainees employ diverse methods to acquire competencies, including self-directed learning through guidelines and textbooks, discussions with peers, and consultations with nurses [2]. Therefore, a variety of educational materials are essential in medical schools to support diverse learning methods and approaches.

At the Department of Anesthesiology, Prince of Songkla University, the training curriculum follows a competency-based design. It emphasizes practical applications, with most trainee activities centered around real-life scenarios and everyday practices to foster the development of diverse competencies. However, certain topics, such as clinical pharmacology, basic anatomy and physiology, and the fundamental principles of general anesthesia, are still taught through traditional lectures. These topics are covered in the first year of training to establish a solid foundation in basic sciences. For example, topics such as non-opioid intravenous anesthetic agents, muscle relaxants and their antagonists, and anesthetic gases and vapors are a part of the Year 1 First-Half Academic Schedule (Supplement 1). Despite this approach, some trainees struggle to translate their theoretical knowledge into clinical practice, highlighting the limitations of a lecture-based teaching model in effectively addressing the learning needs of all trainees.

Active learning methods may be a key that can address these limitations. One such approach is the use of “serious games,” which is effective in increasing participant engagement, fostering learners’ motivation, and promoting knowledge acquisition in a dynamic way. In 1970, Clark Abt defined serious games as those deigned for an explicit and carefully thought-out educational purpose [3]. They are not intended to be played for amusement [3, 4]. Recent literature on serious games has focused on computer-based and digital games [5]. Some studies define serious games as a digital-only tool, which narrows the conceptual scope of the term more than is warranted [4]. Card and board games such as Happy Family [6] and GridlockED [7] fall within the category of serious games when used in medical education. These tabletop games are helpful for memorization, understanding complicated topics, and promoting cognitive processes in low-stakes situations. Their additional advantages include simplicity of preparation, minimal time investment, and the ability to replay for learners [6–8].

In addition to its educational benefits, game-based learning may improve clinical decision-making and positively influence patient care outcomes. A recent scoping review by Edwards et al. emphasized the growing recognition of analog serious games in medical education and highlighted the need for further research into their impact on clinical performance and patient outcomes [9].

In light of these findings, we developed a card game titled “Basic Pharmacology for Anesthesia” as a supplementary learning tool. This study aimed to promote pharmacological knowledge among anesthesiology residents through an engaging and learner-centered format. The card game was evaluated for its effectiveness in teaching first- to third-year anesthesiology residents. Additionally, it aimed to gauge trainees’ satisfaction levels with this game-based learning approach.

## Methods

This cross-sectional observational study was conducted at the Department of Anesthesiology, Faculty of Medicine, Prince of Songkla University. This study was reviewed and approved by the Office of the Human Research Ethics Committee, Faculty of Medicine, Prince of Songkla University, Thailand (approval number REC.66-429-8-1, approval date: November 24, 2023), in accordance with the ethical principles of the Declaration of Helsinki and the International Conference on Harmonization in Good Clinical Practice. Informed consent was obtained from all anesthesiology resident trainees before participating in the study. A clinical trial registry is not applicable to this study.

### Development of card game-based learning and intervention

The card game-based learning was designed as an interactive learning tool, focusing on anesthetic drugs and their pharmacological properties. The list of anesthetic drugs included in the game was determined based on two main criteria: availability within our institution and common usage in clinical practice across Thailand. To keep the gameplay concise and time-efficient, opioids and local anesthetics were excluded from this version and will be considered in the second phase of the study. The contents of the card game were initially reviewed and finalized by the first author and subsequently approved by the co-authors, all of whom are board-certified anesthesiologists. The final draft was then submitted to the School of Medicine and Health Sciences, Faculty of Medicine, Prince of Songkla University, for the production of the official card set.

**Fig. 1:**
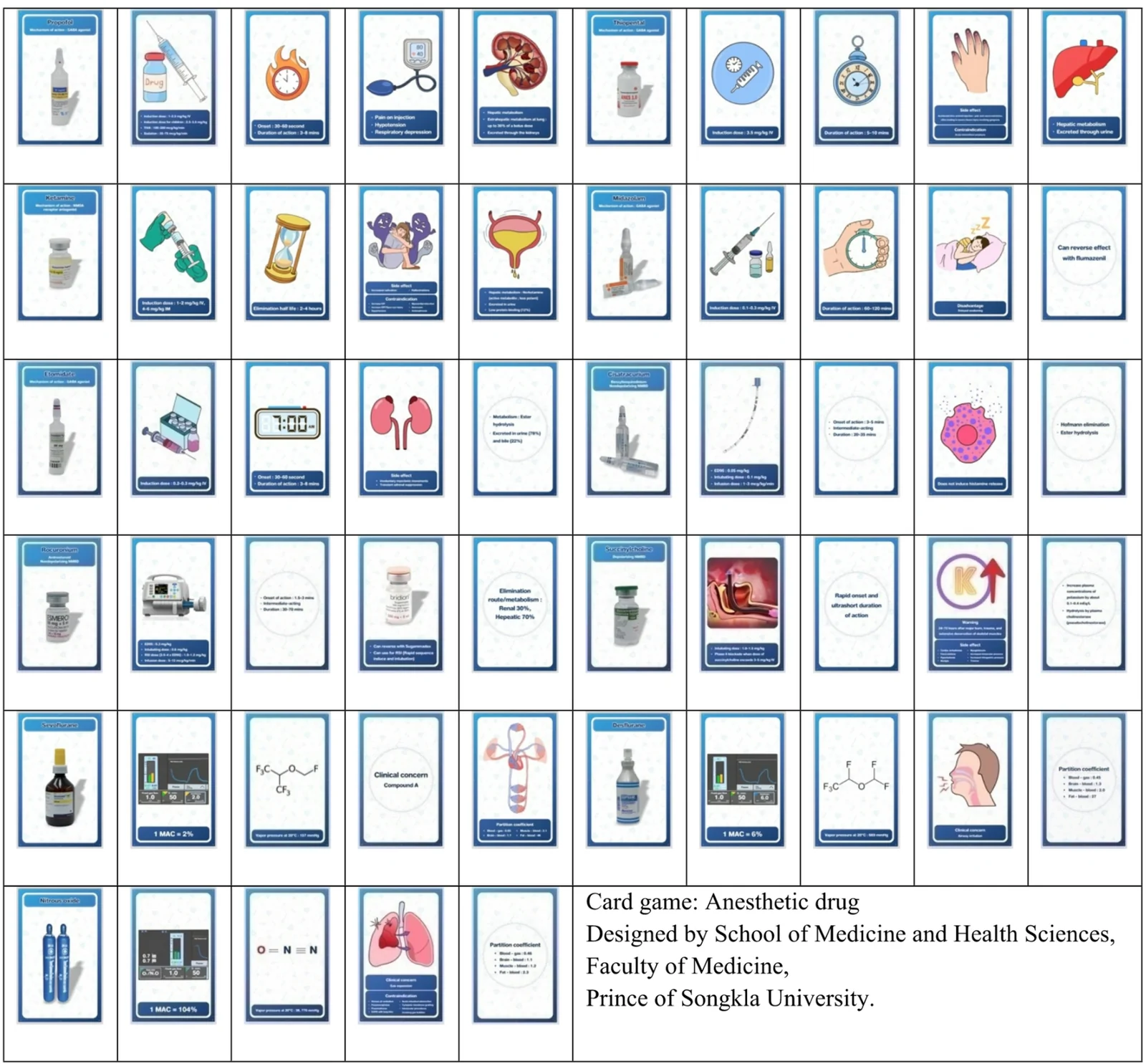
The “Basic Pharmacology for Anesthesia” card game developed by the School of Medicine, Faculty of Medicine, Prince of Songkla University. The educational game consists of 55 cards representing 11 commonly used anesthetic drugs, with each drug represented by five cards containing key pharmacological information

**Fig. 2:**
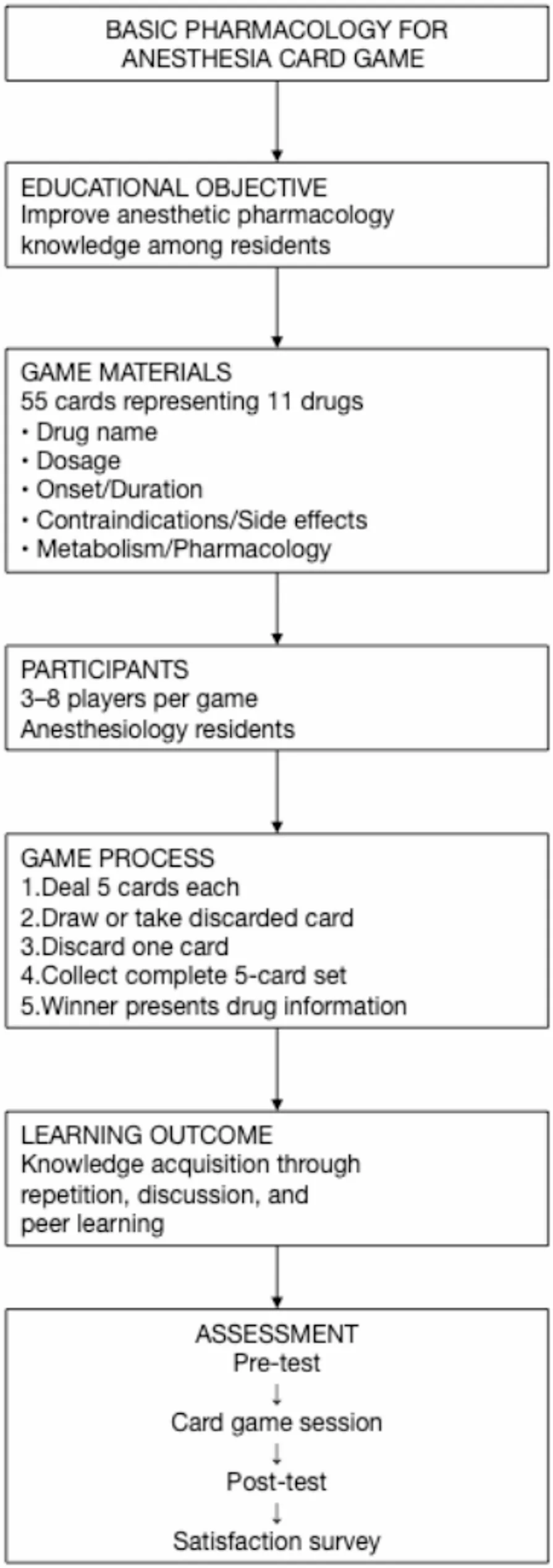
TIDieR description of the “Basic Pharmacology for Anesthesia” card game intervention

The game-based learning was conducted on February 7, 2024. The objective of the game was to collect a complete set of five cards for each specific anesthetic drug. The deck comprised 55 cards representing 11 anesthetic drugs (*propofol*,* thiopental*,* ketamine*,* midazolam*,* etomidate*,* cisatracurium*,* rocuronium*,* succinylcholine*,* sevoflurane*,* desflurane*, and *nitrous oxide*), each of which had cards detailing its name, dosage, onset and duration, contraindications and side effects, and metabolism or pharmacology (Fig. 1). The game accommodated three to eight participants. At the start, the deck was shuffled, and each participant was dealt five cards, with the remaining cards forming the “draw pile” in the center. Participants took turns discarding an unwanted card, passing it to the next participant in a clockwise direction, and drawing one card from the draw pile. Each subsequent participant decided whether to take the discarded card or draw a new one, discarding a card from their hand afterward. The game continued until a participant collected a complete set of cards for one drug, becoming the winner and sharing their set with others to enhance learning about that drug. The game proceeded to determine additional winners using the same process, until only two participants remained. At this point, one final winner was declared, while the remaining was unable to complete a set. Details of the educational intervention, reported according to the Template for Intervention Description and Replication (TIDieR) framework, are provided in Table 1; Fig. 2 [10].

**Table 1:** TIDieR checklist for “Basic Pharmacology for Anesthesia” card game

TIDieR Item	Description
Brief Name	
1.	Basic Pharmacology for Anesthesia Card Game
Why	
2.	To improve anesthesiology residents’ knowledge of anesthetic drug pharmacology through game-based learning.
What	
3.	Materials: Deck of 55 cards representing 11 anesthetic drugs. Each drug contains 5 cards covering: (1) drug name, (2) dosage, (3) onset & duration, (4) contraindications & side effects, and (5) metabolism/pharmacology.
4.	Procedures: Players collect complete 5-card sets for one anesthetic drug. Cards are exchanged by discarding and drawing from a central pile. Winners share the completed set and discuss key pharmacological information with peers.
Who provided	
5.	Developed and validated by four board-certified anesthesiologists from Prince of Songkla University.
How	
6.	Face-to-face tabletop serious game.
Where	
7.	Department of Anesthesiology, Faculty of Medicine, Prince of Songkla University, Thailand.
When and How Much	
8.	Single educational session conducted on February 7, 2024. One round continued until all but one participant completed drug sets.
Tailoring	
9.	Drug selection was tailored to commonly available anesthetic drugs used in Thailand and at the study institution.
Modifications	
10.	No modifications during the intervention.
How well	
11.	Planned: Standardized rules were used for all participants. Same card deck and instructions were used for every session.
12.	Actual: All 19 residents participated using the same game materials and procedures.

### Outcomes

The variables measured in this study included pre- and posttest scores and feedback and satisfaction ratings. The pre- and posttests, conducted before and immediately after the game-based learning, respectively, consisted of identical short-essay questions designed to assess knowledge and the effectiveness of game-based learning. The test was developed by three board-certified anesthesiologists based on key pharmacological concepts outlined in the anesthesiology training curriculum. The participants were instructed to answer the test questions based exclusively on the 11 anesthetic drugs included in the card game. Details of the short-essay questions are provided in Supplement 2. Although certain questions might have had alternate correct responses in broader clinical contexts, the test was intentionally restricted to the content of the game to maintain alignment with the study’s educational objectives. The test had a total score of 20 points, distributed as follows: 8 points for induction drugs, 4 points for volatile anesthetics, and 8 points for neuromuscular drugs. Feedback and satisfaction were assessed across six domains using a five-point Likert scale, ranging from 1 to 5 (5 = very satisfied, 4 = satisfied, 3 = neutral, 2 = dissatisfied, and 1 = very dissatisfied). Although the comments were recorded in free-text format, thematic grouping was applied to categorize the responses.

### Sample size

The sample size was calculated based on the formula used to test one population mean:

$$n=\frac{\left(z_{1-\frac{a}{2}}+z_{1-\beta}\right)^{2}\sigma^{2}}{\left(\mu-\mu_{0}\right)^2}$$

A previous study investigating the use of a serious game to teach medical students about cleft lip and palate treatment a pretest mean (standard deviation [SD]) score of 12.23 (2.21) and a posttest mean (SD) score of 14.43 (1.66) out of 17 points [18]. The sample size for testing one population mean was calculated using the n4Studies Plus application [11–13].

The output for a two-tailed test included the following parameters:

$$\mathrm{Z}\left(0.975\right)=1.96, \mathrm{Z}\left(0.8\right)=0.84$$

(Reference value (µ0) = 12.23, Mean (µ) = 14.43, Standard deviation (σ) = 1.66)

Based on these inputs, the calculated sample size was *n* = 4.46. Rounding up, the final required sample size was determined to be 5.

### Statistical analysis

Categorical variables were reported as frequency and percentages. All continuous variables were tested for the normal distribution and were presented as either mean and standard deviation (SD) or median and interquartile range (IQR), as appropriate. Paired Continuous variables were analyzed using the Wilcoxon signed-rank test. A *p*-value of less than 0.05 was considered statistical significance. Data management and analysis were performed using R software, version 4.3.3.

## Results

Nineteen resident trainees participated in the study (8/19 [42.1%] first-year residents, 4/19 [21.1%] second-year residents, and 7/19 [36.8%] third-year residents; 8/19 [42.1%] male residents) (Supplement 3). Comparison of pre- and posttest scores revealed improvements in all categories: 5.00 vs. 7.47 (*p* < 0.001) for induction drugs, 2.59 vs. 3.92 (*p* < 0.001) for volatile anesthetics, and 6.67 vs. 7.51 (*p* < 0.001) for neuromuscular agents. Additionally, the total score significantly increased from 14.26 (pretest) to 18.91 (posttest) (*p* < 0.001) (Table 2). The maximum possible total score was 20 points.

**Table 2:** Comparison of knowledge scores before and after the game-based learning intervention. Data are presented as mean (SD). Maximum possible scores for each domain are shown in parentheses

Knowledge domain(maximum score)	Pretest, mean (SD)	Posttest, mean (SD)	*p*-value
Induction drug (8)	5.00 (1.18)	7.47 (0.66)	<0.001
Volatile anesthetics (4)	2.59 (0.69)	3.92 (0.25)	<0.001
Neuromuscular agent (8)	6.67 (0.79)	7.51 (0.54)	<0.001
Total score (20)	14.26 (1.55)	18.91 (1.05)	<0.001

*Abbreviation*: *SD *standard deviation

**Fig. 3:**
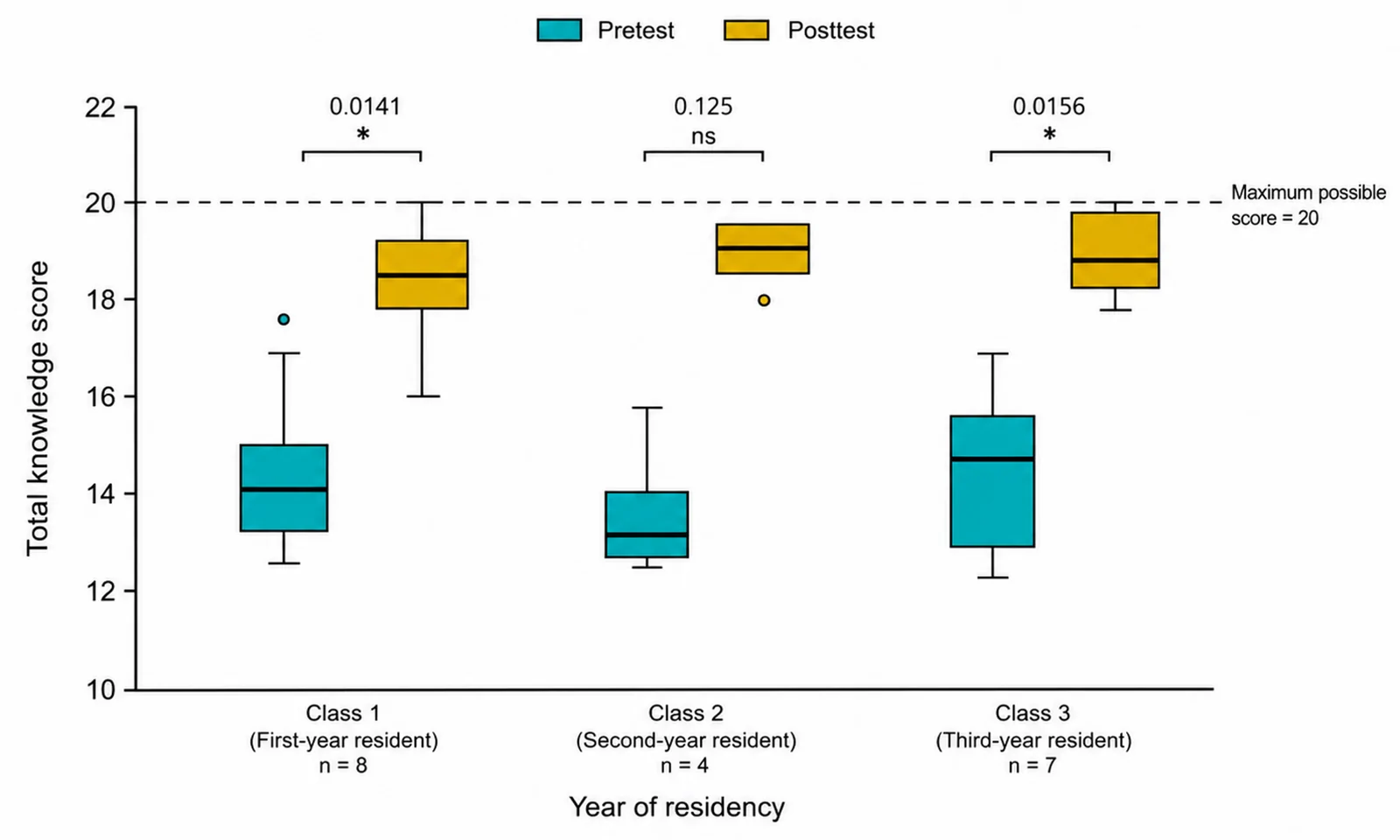
Comparison of pretest and posttest total knowledge scores, classified by year of residency. Numbers above the brackets indicate *p*-values from paired comparisons (Wilcoxan signed-rank test). *: *p*-value < 0.05. Scores are presented as box plots showing median (center line), interquartile range (box). Outliers are shown as individual dots. Maximum possible total score = 20 points. ns = not statistically significant (*p*-value > 0.05)

Subgroup analysis by year of residency revealed that second-year residents showed no significant difference between pre- and posttest scores, whereas first- and third-year residents demonstrated significant improvements (Fig. 3), (Supplement 4). The level of knowledge gained through game-based learning was rated 4.84 (0.37), and the application of this knowledge was rated 4.95 (0.23). The appropriateness of the game format was rated 4.84 (0.37), and the feasibility of playing the game was rated 4.68 (0.48). The optimal number of cards for the game was rated 4.42 (0.69), and the overall satisfaction level was 4.84 (0.37) (Table 3).

**Table 3:** Participant feedback and satisfaction scores following the game-based learning intervention (*n* = 19). Scores were assessed using a 5-point Likert scale, where 1 = very dissatisfied, 2 = dissatisfied, 3 = neutral, 4 = satisfied, and 5 = very satisfied. Data are presented as mean (SD)

Feedback item	Mean (SD)	Min	Max
1. How much knowledge have you gained from playing the game-based learning?	4.84 (0.37)	4	5
2. How much of the knowledge you gained can be applied in clinical practice?	4.95 (0.23)	4	5
3. Is the format of the game appropriate?	4.84 (0.37)	4	5
4. Is the level of difficulty or ease of playing the game suitable for the player?	4.68 (0.48)	4	5
5. Are the number of cards to play the game optimal?	4.42 (0.69)	3	5
6. What is the level of overall satisfaction?	4.84 (0.37)	4	5
Total satisfaction score (maximum score = 30)	28.58 (1.98)	23	30

*Abbreviations*: *SD *standard deviation

Residents made several suggestions to enhance game-based learning. First, the residents recommended to limit the number of players per round to optimize the gameplay. Second, displaying card details to all players was suggested to facilitate learning. Third, ensuring that the images on the cards correspond with the data would improve recall. Some feedback highlighted issues such as small font size, thinness of the cards, and their oversized dimensions, which made them difficult to handle.

## Discussion

Our study demonstrated that a serious analog game significantly improved anesthesiology residents’ knowledge of anesthetic drugs, as evidenced by a statistically significant increase in total test scores and scores across multiple drug categories. This magnitude of improvement is consistent with the findings of other studies that explored serious games in medical education. For instance, Muthiyan et al. reported statistically significant gains in factual knowledge among medical students following a pharmacology card game intervention [8].

In our study, the resident satisfaction was high, which aligns with the positive learner reception of analog serious games reported in prior literature on GridlockED and Happy Family [6, 7]. Despite these encouraging results, the knowledge quiz may have limitations in terms of validity, as some questions could allow for alternative correct responses. Additionally, our study lacked a control group for comparison with traditional lecture-based education, which limited our ability to directly compare learning outcomes.

In our institution, lectures related to card games were delivered in August during the first year of residency training, whereas our study was conducted in February. Despite the relatively recent instruction through lectures, first-year residents still had low pretest scores. This finding suggests that game-based learning can help students review and reinforce their knowledge. Additionally, third-year residents showed a significant difference between pretest and posttest scores, indicating that they may have forgotten some fundamental concepts of anesthetic pharmacology.

Notably, the pre- and posttest scores were similar across all years of residency training. This finding suggests that traditional lectures do not lead to strong knowledge retention. One possible reason is that first-year residents attended more lectures (*n* = 31) than second- and third-year residents (*n* = 11 and *n* = 13, respectively) (Supplement 1). Another possible explanation is that first-year lectures focused on basic knowledge, whereas the second- and third-year lectures emphasized the application of that knowledge in clinical practice.

The game-based learning approach in our study utilized a serious game in which players actively engaged with the game to acquire knowledge. This contrasts with gamification, which incorporates gaming elements as only one part of the learning process [14]. Serious games offer numerous benefits, including promoting active participation, enhancing problem-solving abilities, improving clinical reasoning and decision-making skills, and encouraging teamwork [15–17]. Our findings suggest that analog serious games are a valuable and engaging educational strategy that can supplement conventional teaching, especially in reinforcing pharmacology knowledge among anesthesiology trainees. Given their simplicity and low cost, these tools may be particularly beneficial in resource-limited settings. We designed the intervention as a card game owing to its simplicity and shorter development time compared to digital games. However, serious games can be designed in various formats, including card, board, and digital games, depending on the intended objectives and available resources [4].

The outcomes for game-based learning evaluated in our study were knowledge gain and satisfaction. However, the evaluation of learning management using game-based learning can encompass various aspects, including assessing the effectiveness of games in enhancing learners’ knowledge [18–21], evaluating satisfaction with game-based learning activities [19, 22], measuring learner engagement [23, 24], and comparing the outcomes of game-based learning with those of traditional teaching methods [25]. To achieve a more comprehensive evaluation of the educational impact, future research should incorporate other assessment methods, compare serious games with standard educational formats, assess long-term knowledge retention, and further evaluate the validity of assessment tools. A recent scoping review by Edwards et al. [9] highlighted these areas as key priorities for future investigations.

## Limitations

Our study has some limitations. First, the sample size was limited to anesthesiology resident trainees, which may have restricted the generalizability of the findings. To address this limitation, future studies should include participants from other institutions or adopt a multicenter approach. Second, the study was conducted under time constraints. The posttest was administered immediately after the game-based learning session, primarily to assess short-term memory. This study did not evaluate or provide insights into long-term memory retention, which should be explored in future research. Third, the measurement tools used, such as short-answer questions, may not fully capture the knowledge gained from game-based learning methods. Future studies should employ a more comprehensive assessment framework to better evaluate knowledge acquisition. Finally, the scope of the study was confined to commonly available anesthetic drugs at our institution. Thus, certain drug categories, such as other induction agents, neuromuscular blocking drugs, and volatile anesthetics, were excluded. These drugs can be included in future studies to provide a more comprehensive understanding of the educational impact.

## Conclusion

Game-based learning can enhance anesthesiology residents’ understanding of anesthetic drugs and has shown high levels of learners’ satisfaction. To achieve a more comprehensive evaluation of this educational intervention, future studies should include additional assessment methods, such as measuring learner engagement and comparing game-based learning with traditional methods used to teach anesthetic drugs to anesthesiology residents.

## Supplementary Information

Supplementary Material 1.

Supplementary Material 2.

Supplementary Material 3.

## Data Availability

The raw datasets used and/or analyzed during the current study are available in the related files of the submission. Details of the game card are provided in the supplementary section.
