# Supplementary material for: Effectiveness of game-based learning of anesthetic drugs in anesthesiology residents: a pilot study
Source: BMC Med Educ. 2026 Jul 27;26:1700. doi: 10.1186/s12909-026-10011-5 (PMC13689327; doi:10.1186/s12909-026-10011-5)
Supplement: Supplementary file 1 [file 12909_2026_10011_MOESM1_ESM.pdf]

CARD GAME

ANESTHETIC DRUG

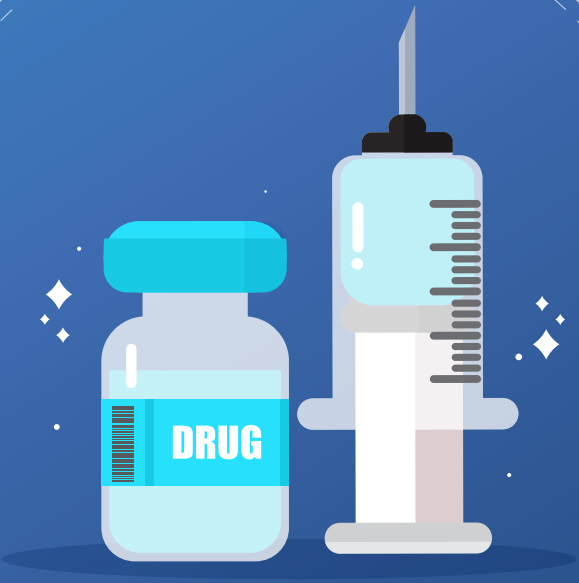

PSU FACULTY OF MEDICINE | School of Medicine and Health Sciences

Propofol

Mechanism of action : GABA agonist

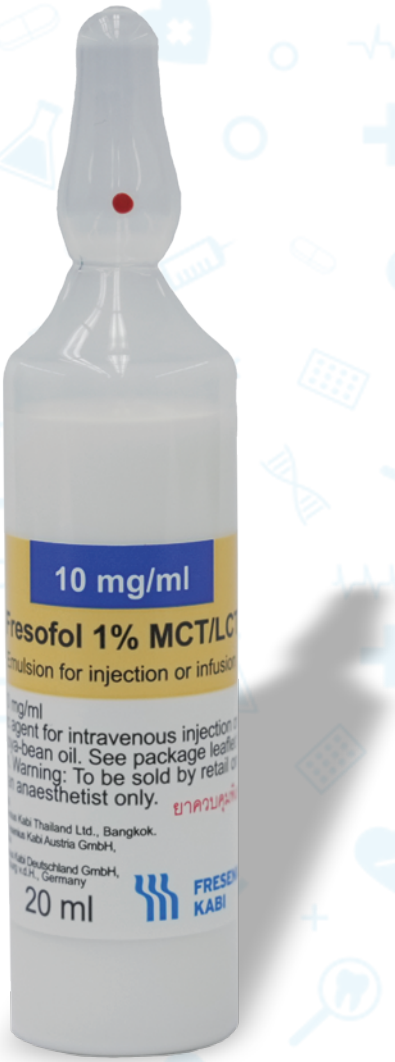

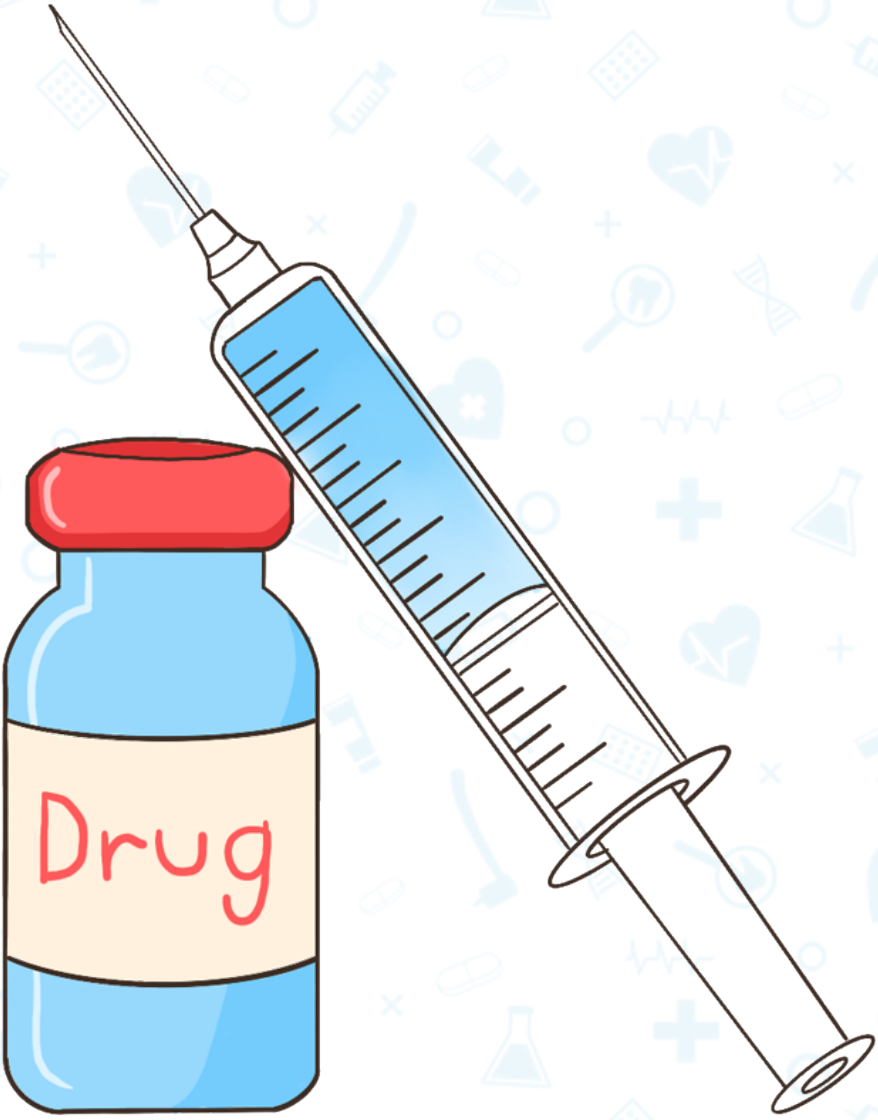

- Induction dose : 1-2.5 mg/kg IV
- Induction dose for children : 2.5-3.5 mg/kg
- TIVA : 100-200 mcg/kg/min
- Sedation : 25-75 mcg/kg/min

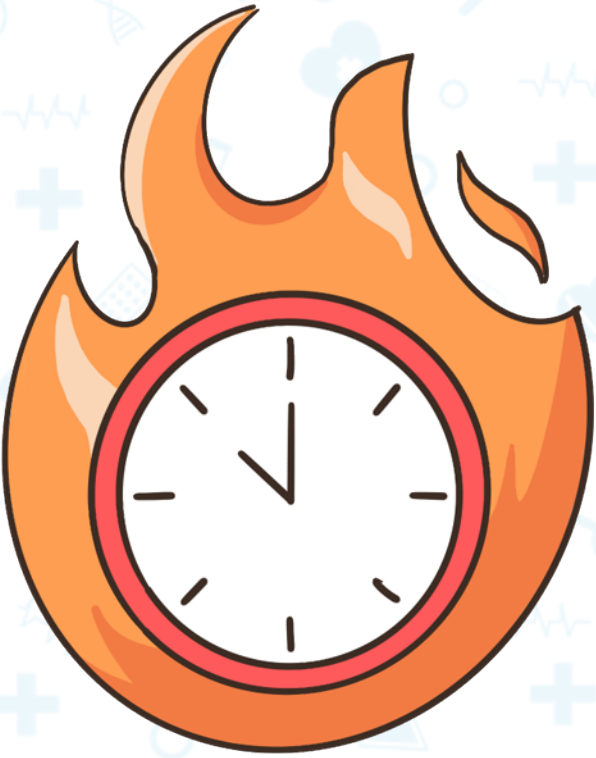

- Onset : 30-60 second
- Duration of action : 3-8 mins

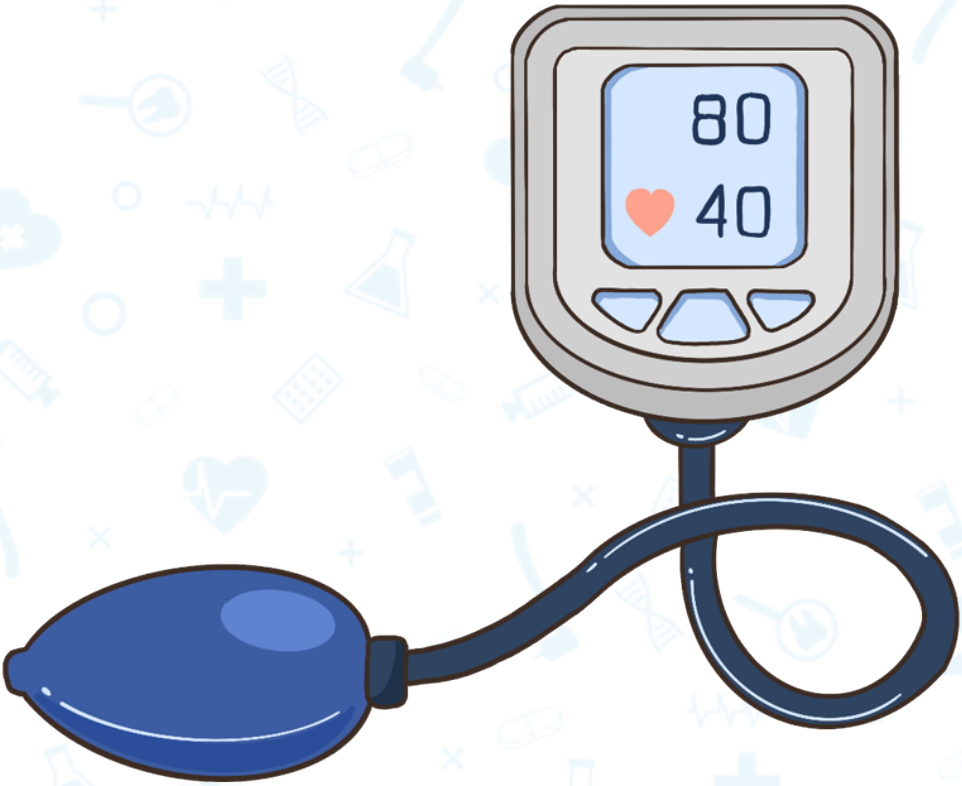

- Pain on injection
- Hypotension
- Respiratory depression

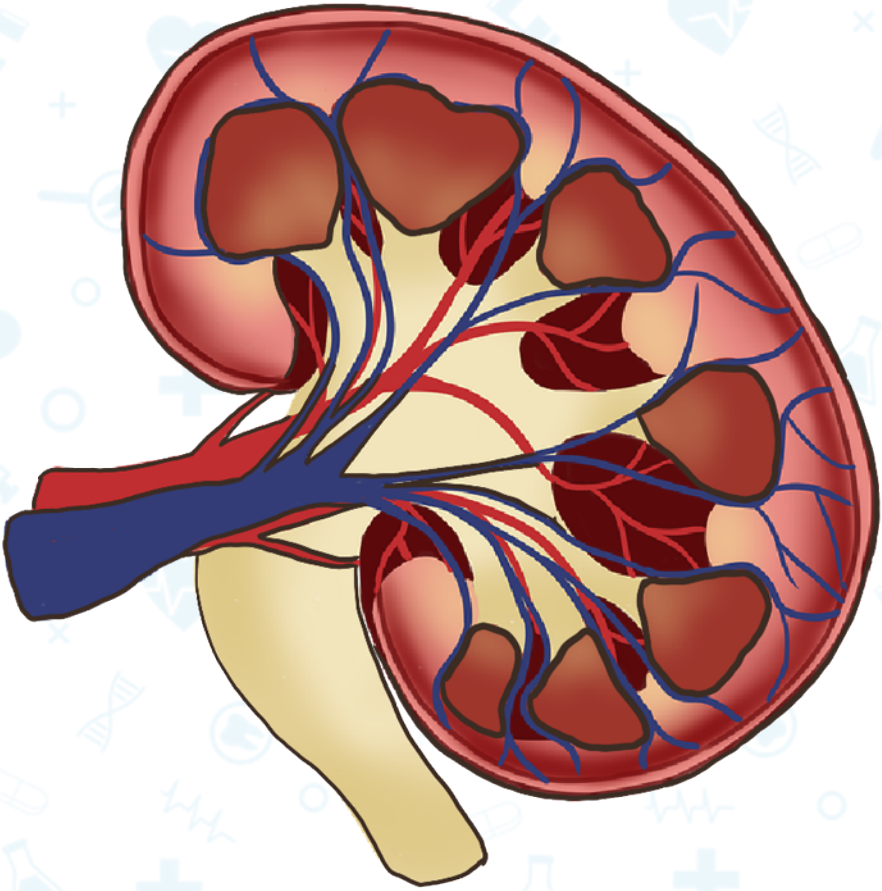

- Hepatic metabolism
- Extrahepatic metabolism at lung : up to 30% of a bolus dose
- Excreted through the kidneys

Thiopental

Mechanism of action : GABA agonist

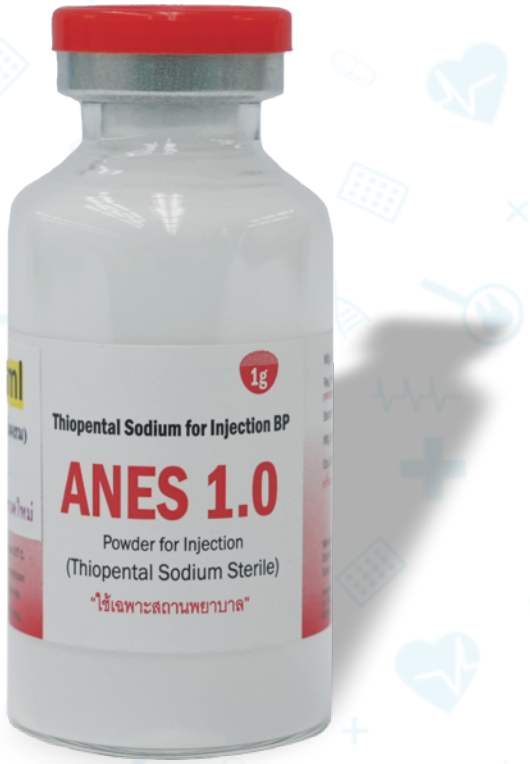

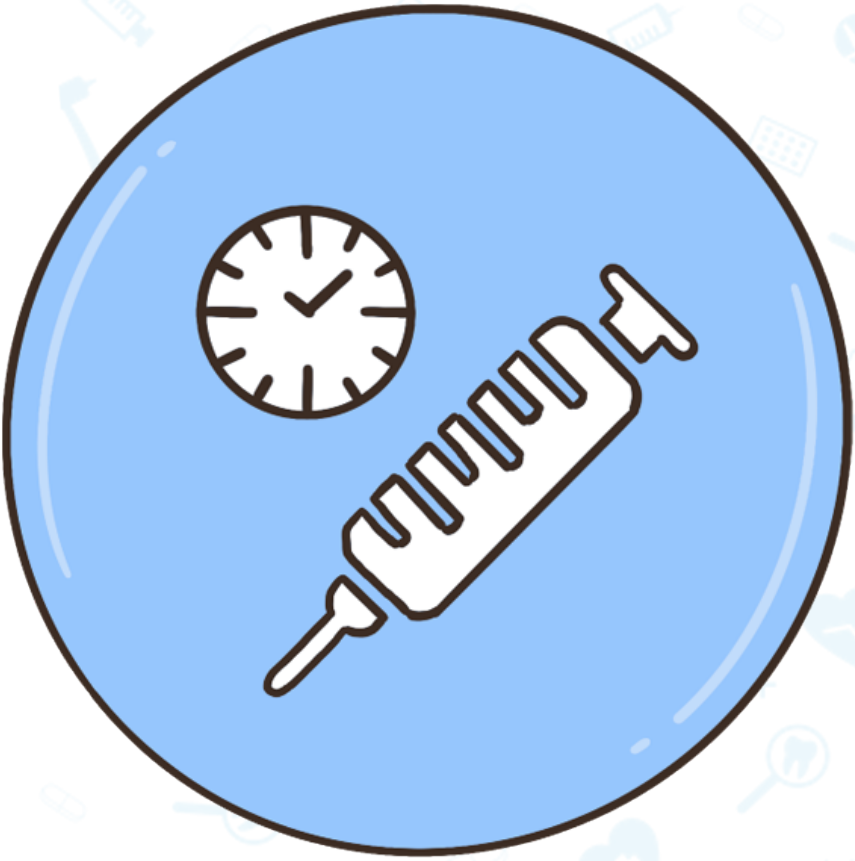

Induction dose : 3.5 mg/kg IV

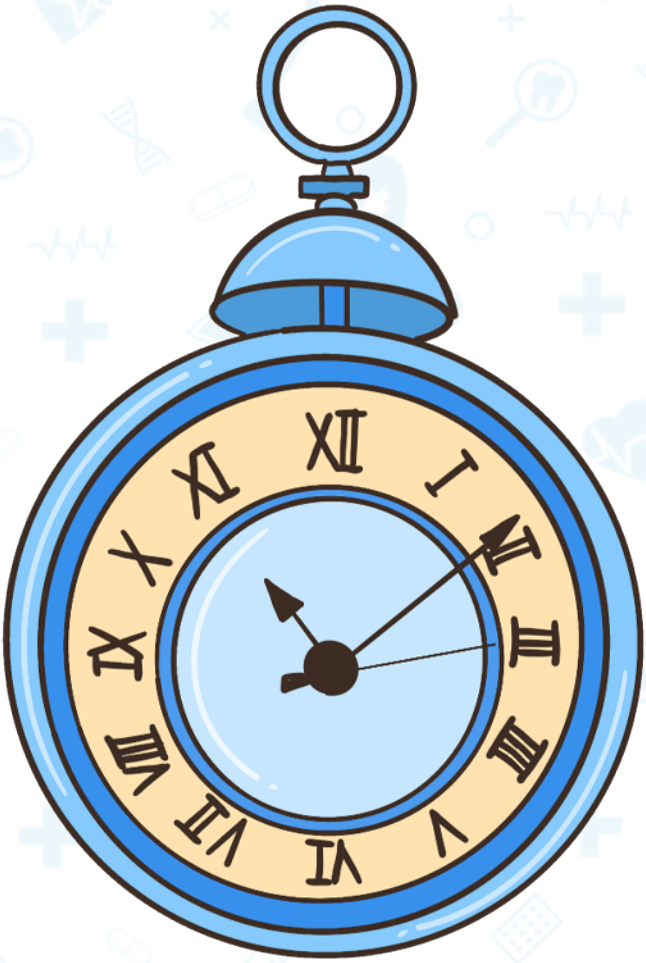

Duration of action : 5–10 mins

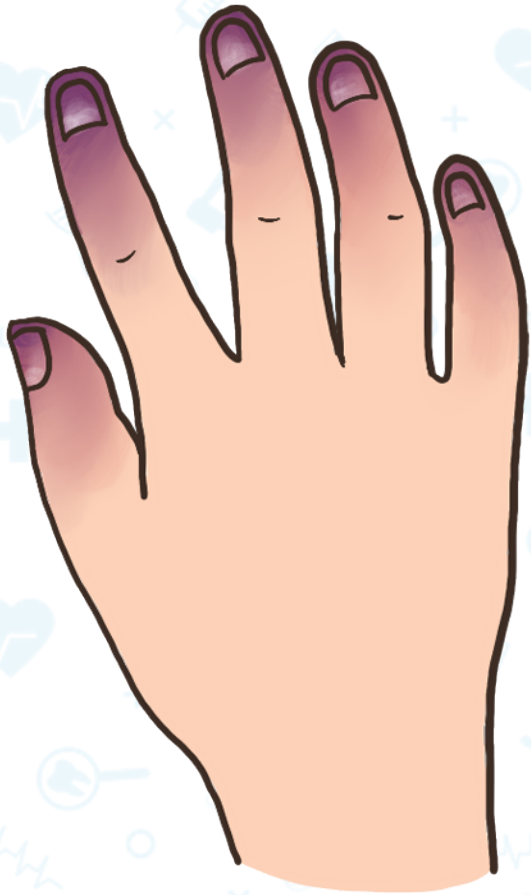

Side effect

Accidental intra-arterial injection : pain and vasoconstriction, often leading to severe tissue injury involving gangrene

Contraindication

Acute intermittent porphyria

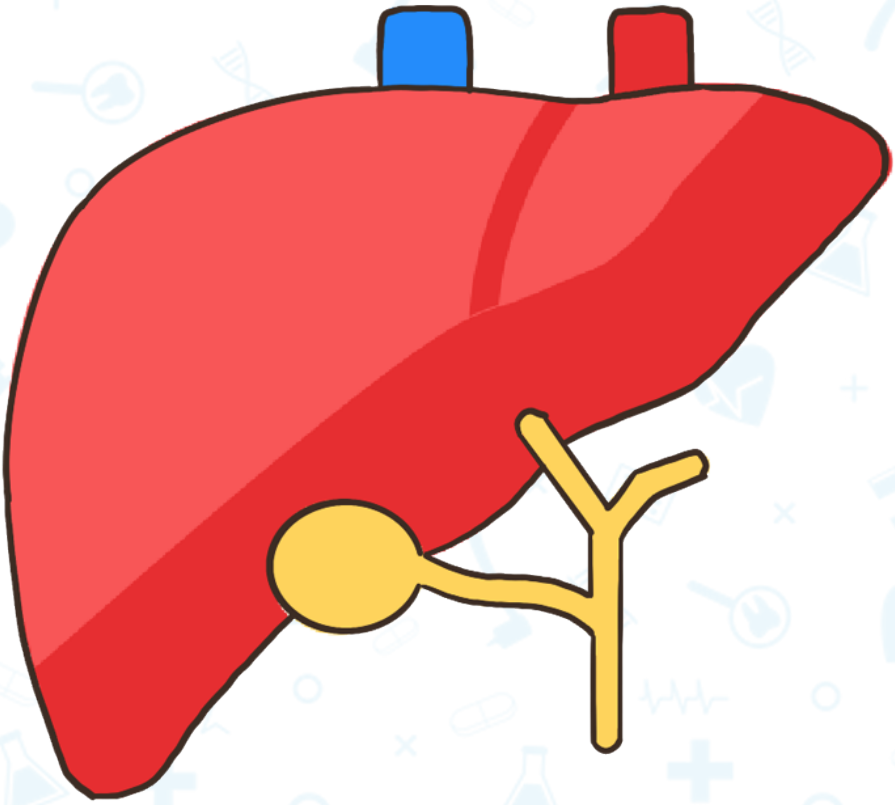

- Hepatic metabolism
- Excreted through urine

Ketamine

Mechanism of action : NMDA receptor antagonist

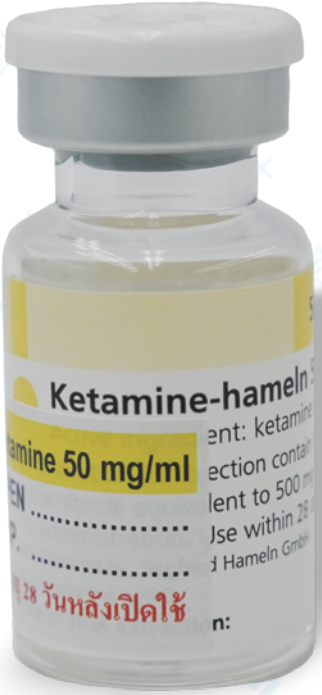

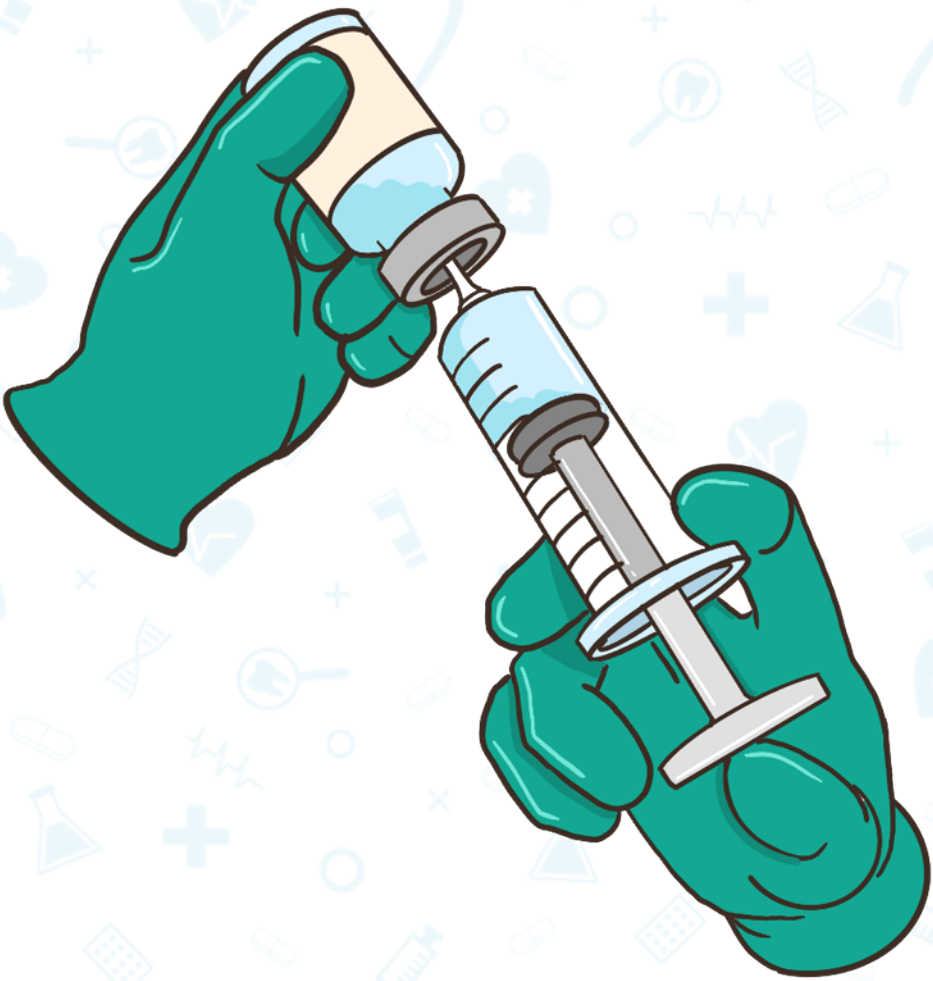

Induction dose : 1–2 mg/kg IV,
4–6 mg/kg IM

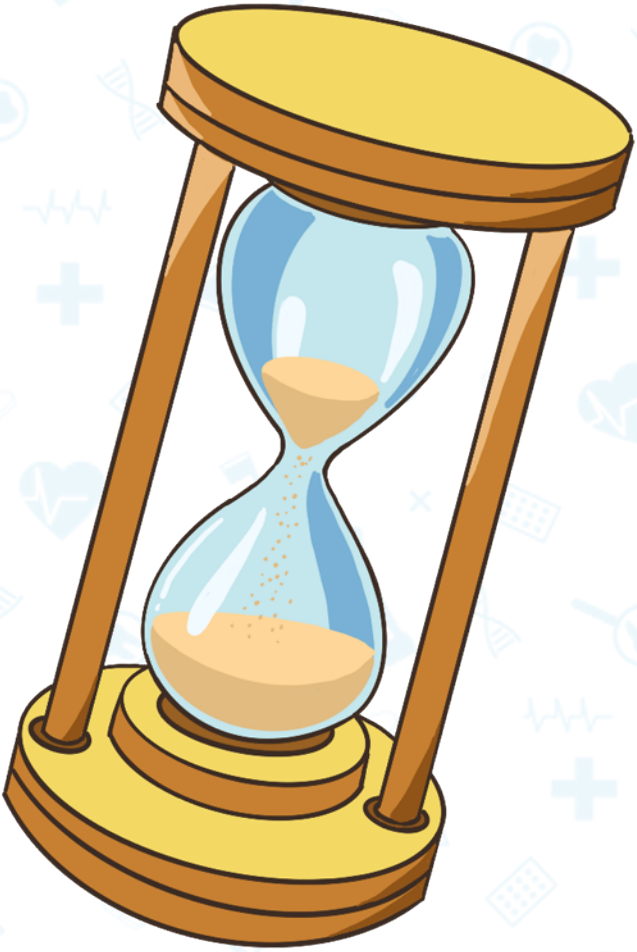

Elimination half life : 2-4 hours

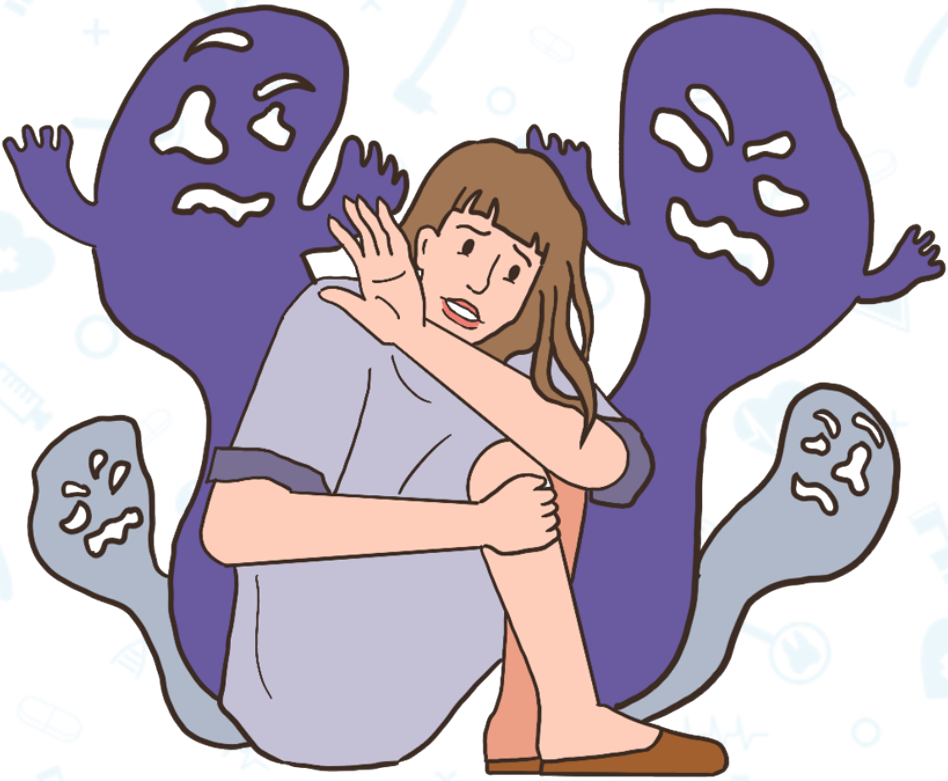

Side effect

- Increased salivation
- Hallucinations

Contraindication

- Increase ICP
- Increase IOP/Open eye injury
- Hypertension
- Myocardial infarction
- Aneurysm
- Schizophrenia

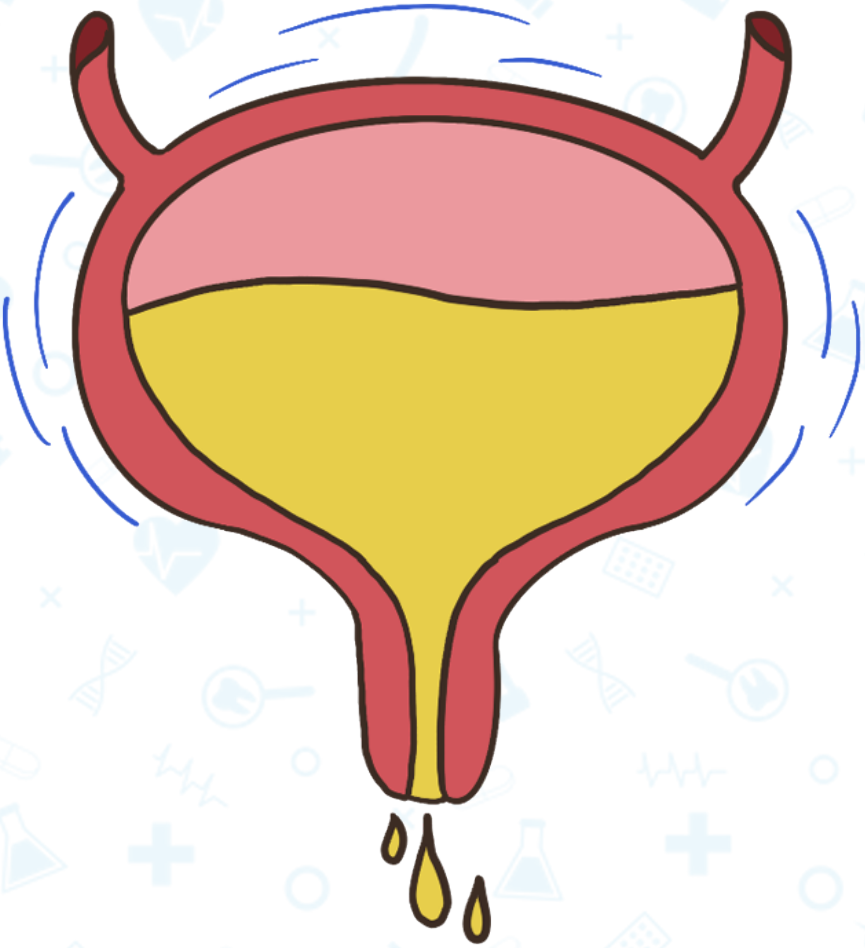

- Hepatic metabolism : Norketamine (active metabolite ; less potent)
- Excreted in urine
- Low protein binding (12%)

Midazolam

Mechanism of action : GABA agonist

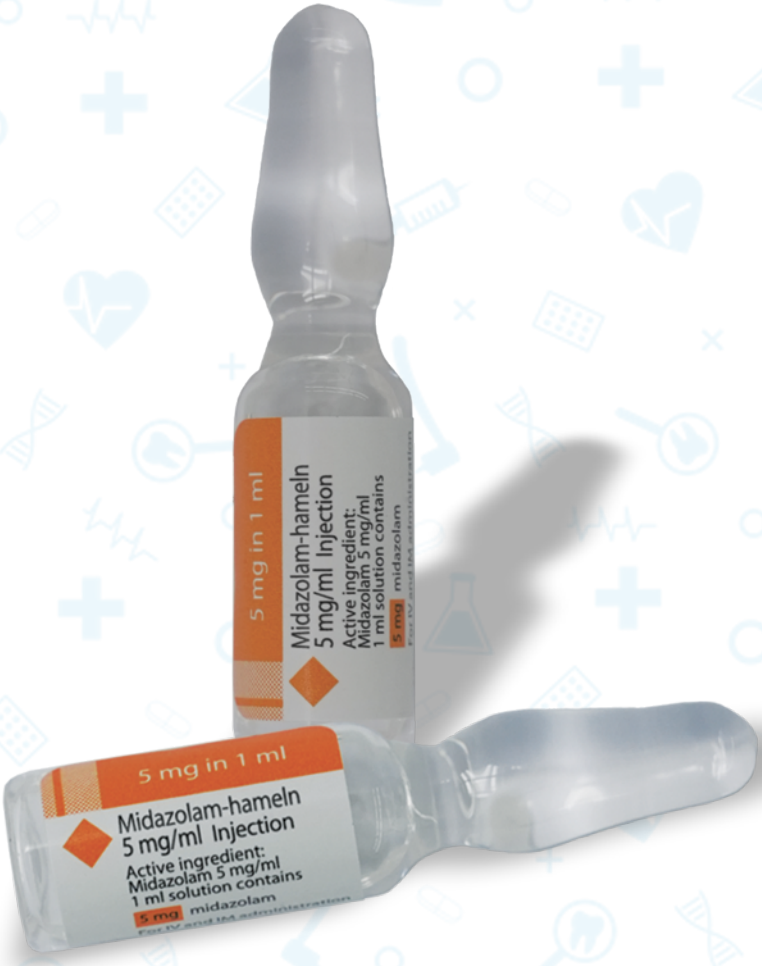

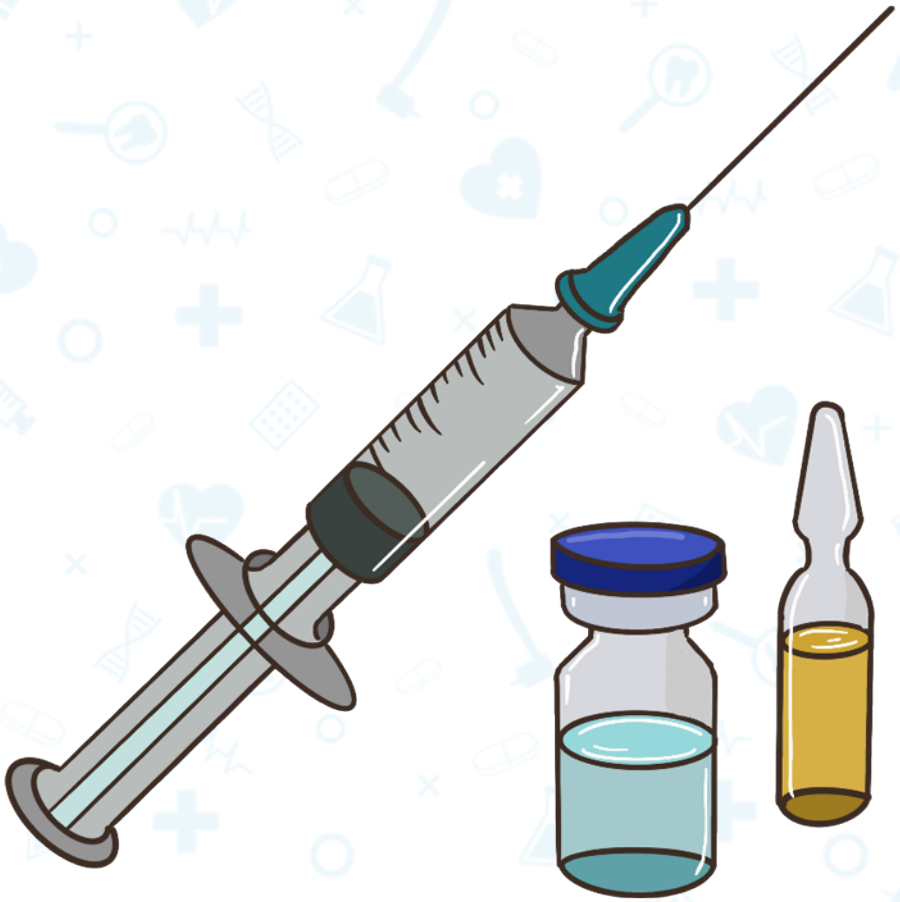

Induction dose : 0.1–0.3 mg/kg IV

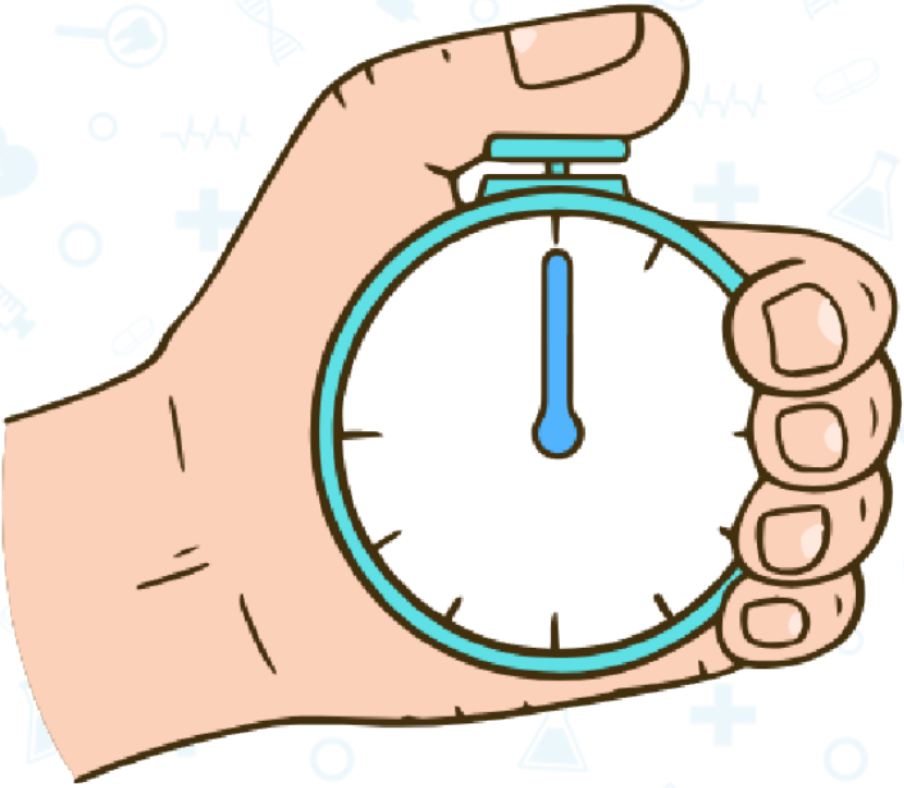

Duration of action : 60-120 mins

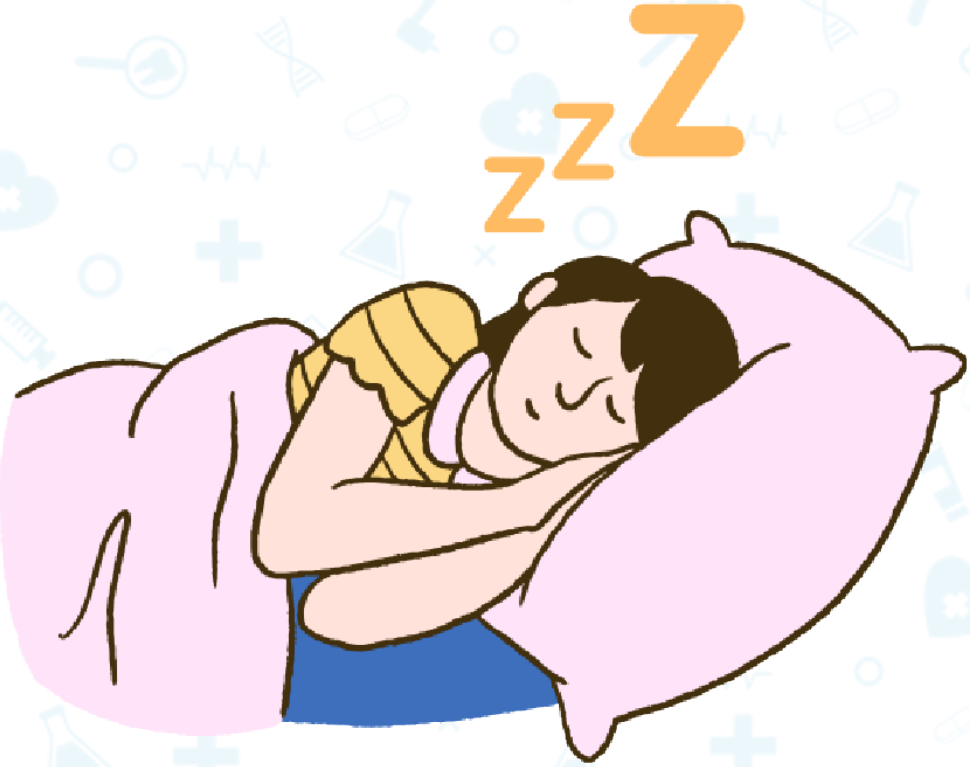

Disadvantage

Delayed awakening

Can reverse effect with flumazenil

Etomidate

Mechanism of action : GABA agonist

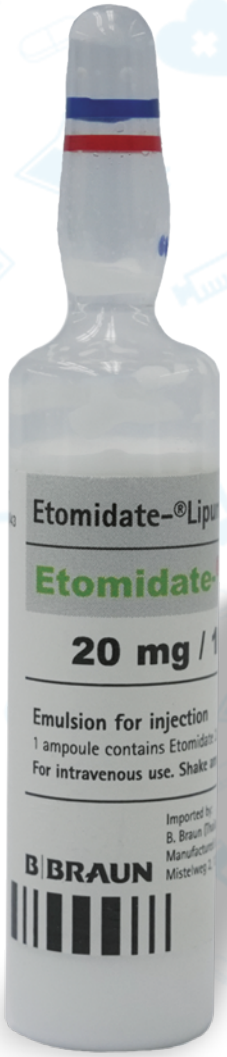

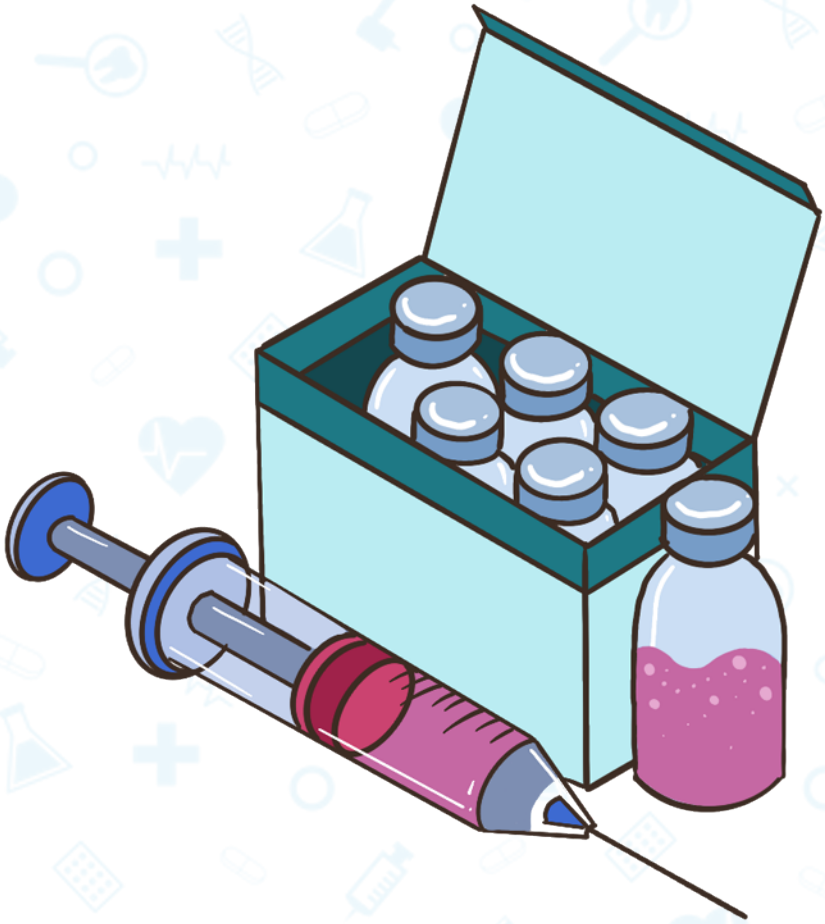

Induction dose : 0.2-0.3 mg/kg IV

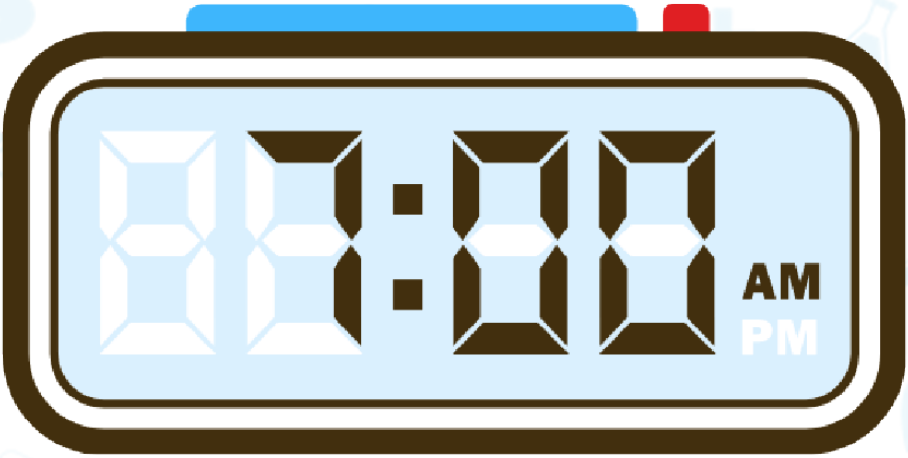

- Onset : 30–60 second
- Duration of action : 3–8 mins

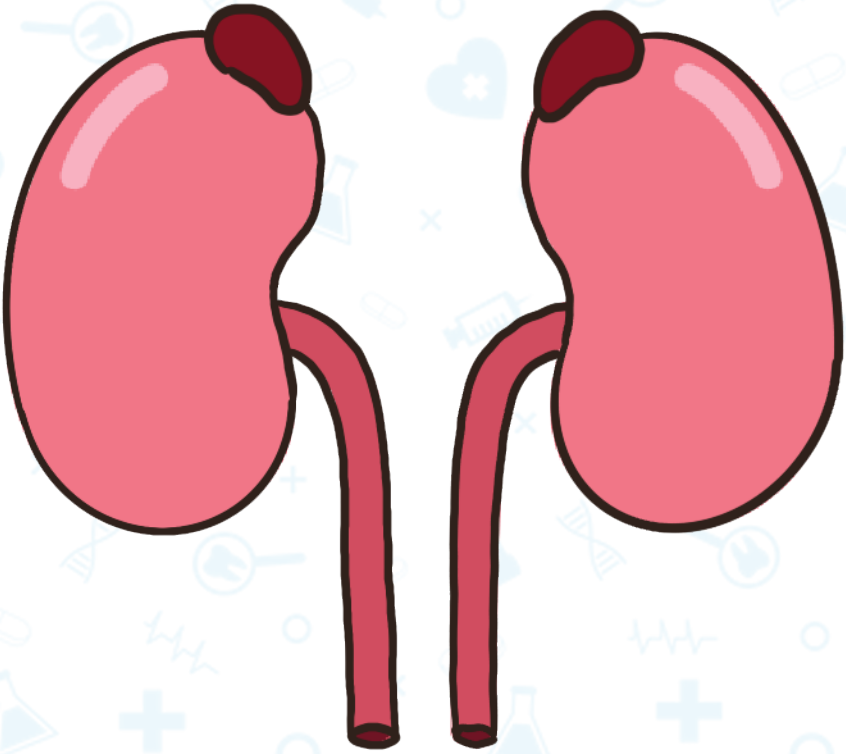

Side effect

- Involuntary myoclonic movements
- Transient adrenal suppression

- Metabolism : Ester hydrolysis
- Excreted in urine (78%) and bile (22%)

Cisatracurium

Benzylisoquinolinium
Nondepolarizing NMBD

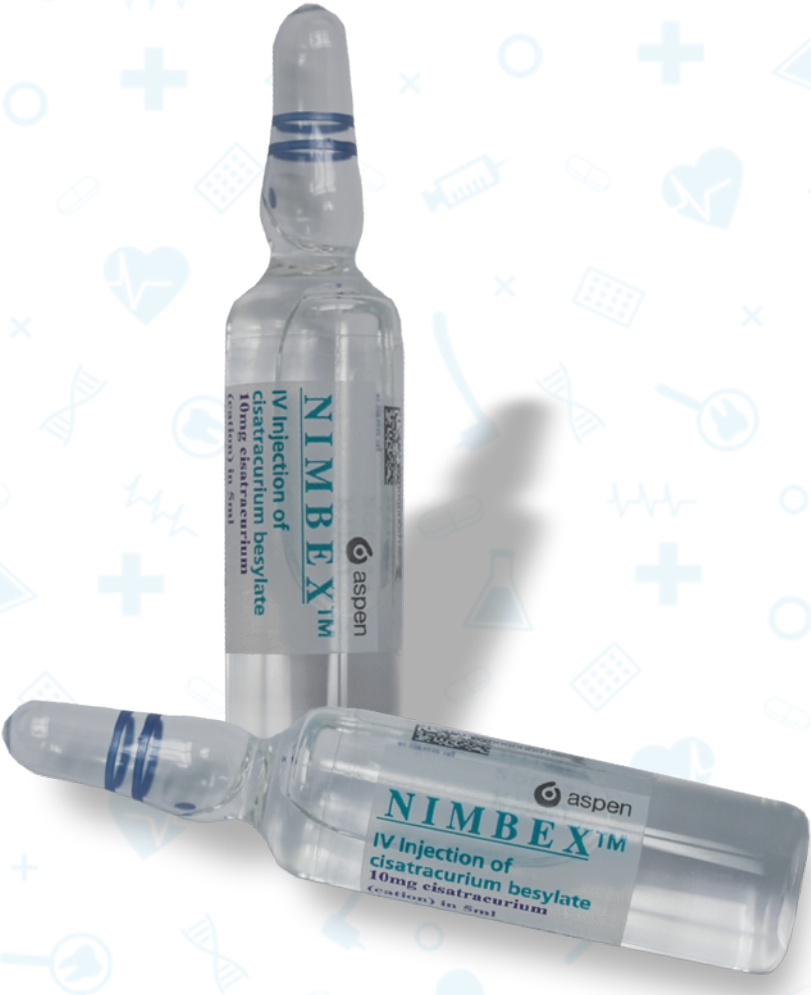

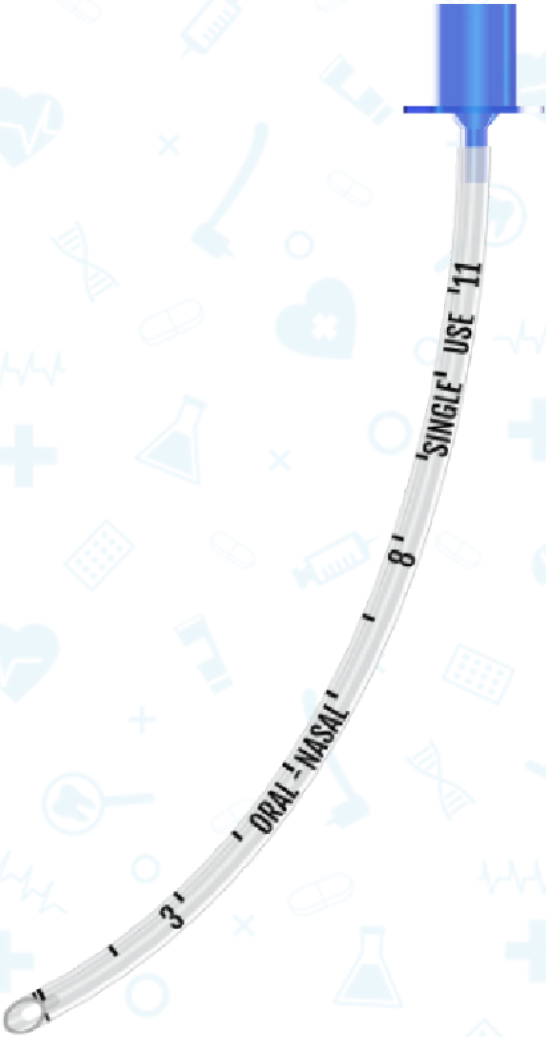

- ED95 : 0.05 mg/kg
- Intubating dose : 0.1 mg/kg
- Infusion dose : 1-3 mcg/kg/min

- Onset of action : 3–5 mins
- Intermediate-acting
- Duration : 20–35 mins

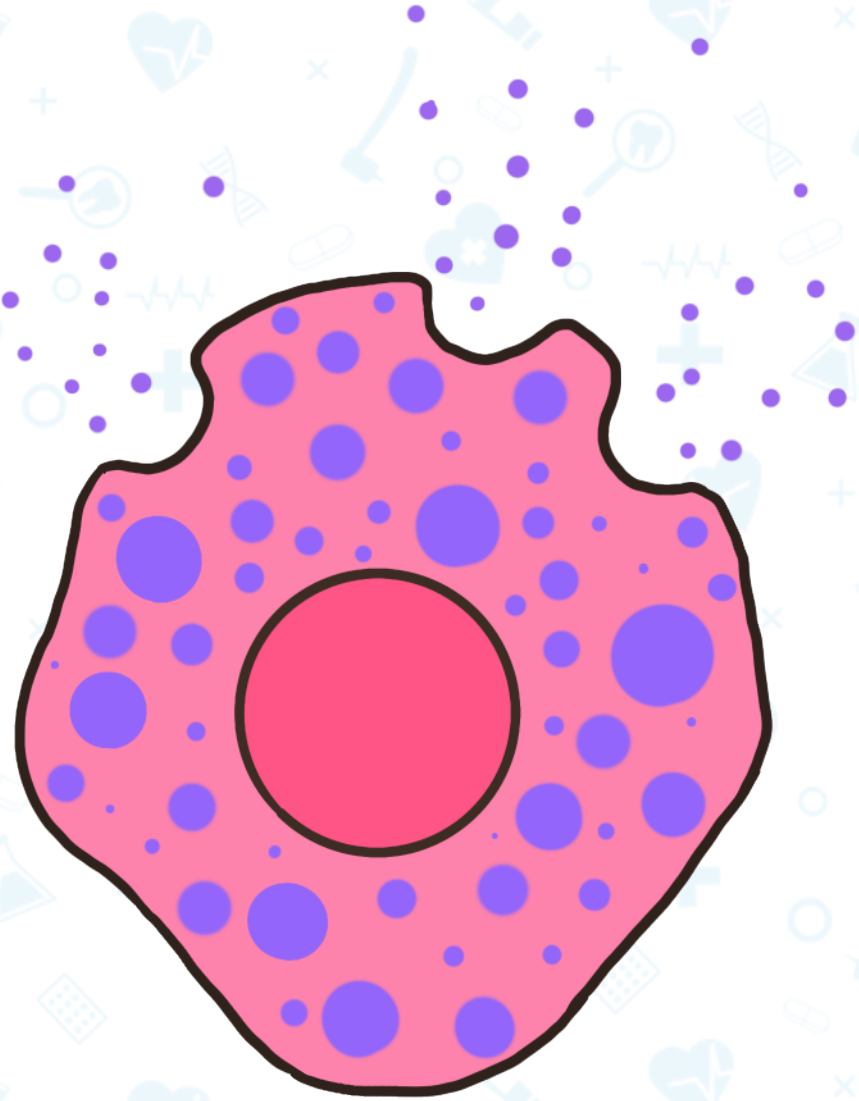

Does not induce histamine release

- Hofmann elimination
- Ester hydrolysis

Rocuronium

Aminosteroid
Nondepolarizing NMBD

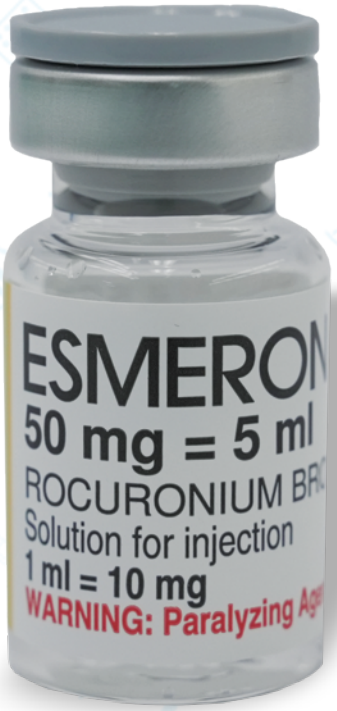

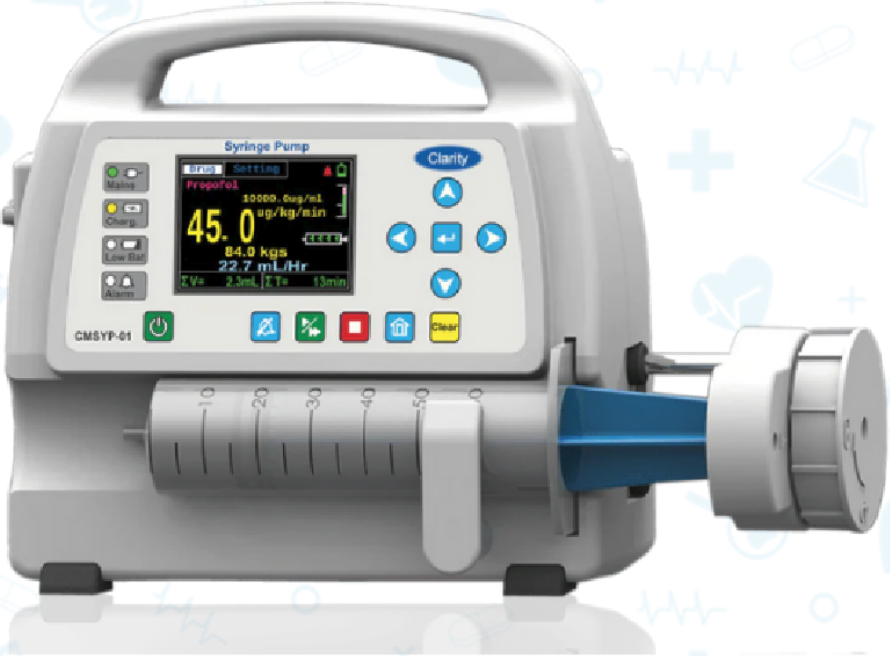

- ED95 : 0.3 mg/kg
- Intubating dose : 0.6 mg/kg
- RSI dose (3.5–4 x ED95) : 1.0–1.2 mg/kg
- Infusion dose : 5–12 mcg/kg/min

- Onset of action : 1.5-3 mins
- Intermediate-acting
- Duration : 30-70 mins

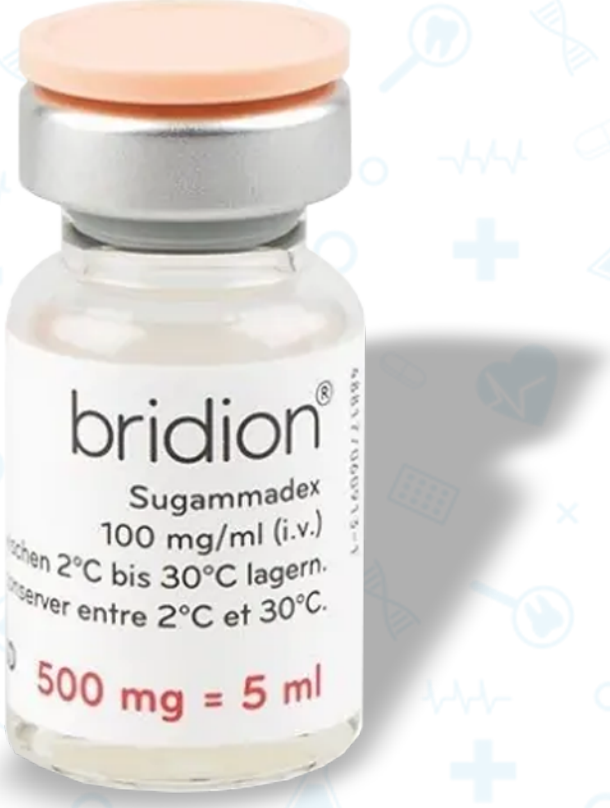

- Can reverse with Sugammadex
- Can use for RSI (Rapid sequence induce and intubation)

Elimination route/metabolism : Renal 30%, Hepeatic 70%

Succinylcholine

Depolarizing NMBD

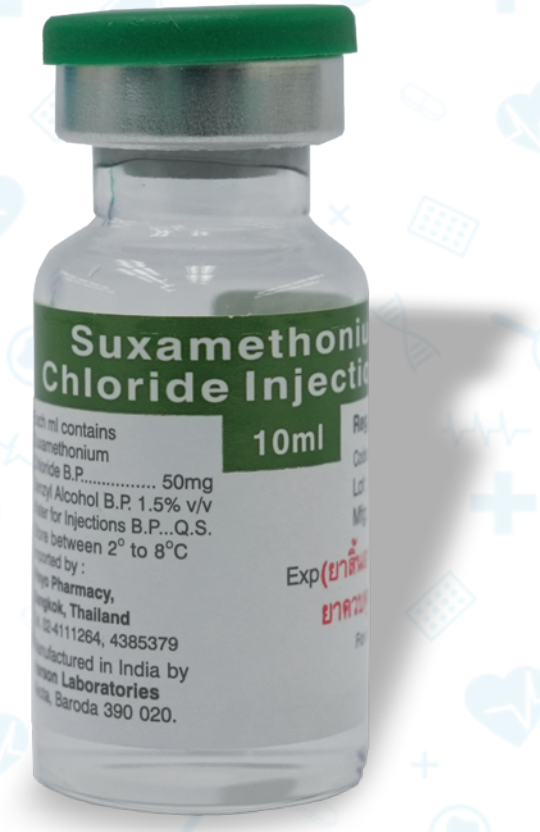

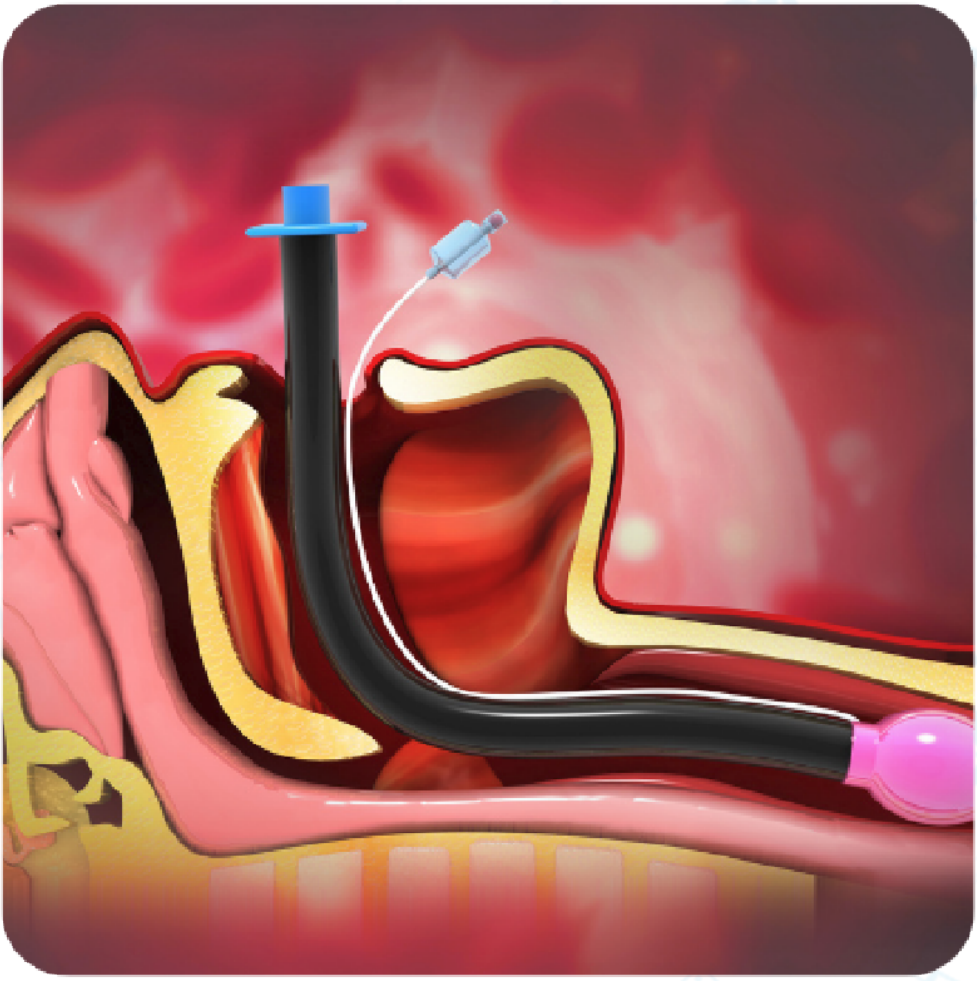

- Intubating dose : 1.0-1.5 mg/kg
- Phase II blockade when dose of succinylcholine exceeds 3-5 mg/kg IV

Rapid onset and ultrashort duration of action

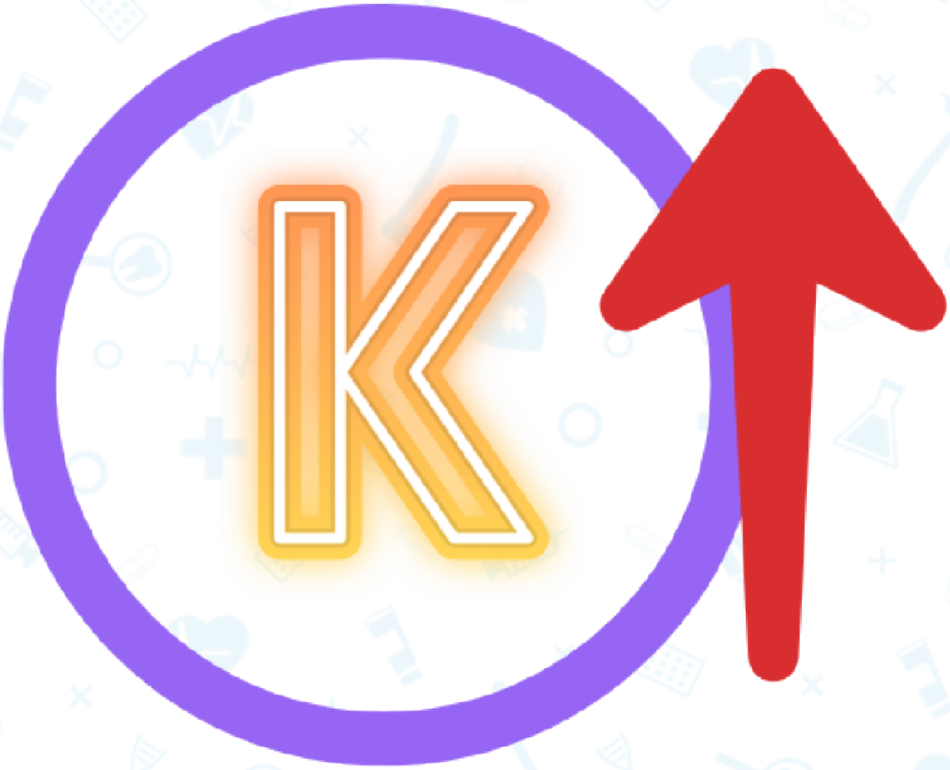

Warning

24–72 hours after major burn, trauma, and extensive denervation of skeletal muscles

Side effect

- Cardiac arrhythmias
- Fasciculations
- Hyperkalemia
- Myalgia
- Myoglobinuria
- Increased intraocular pressure
- Increased intragastric pressure
- Trismus

- **Increase plasma concentrations of potassium by about 0.1-0.4 mEq/L**
- **Hydrolysis by plasma cholinesterase (pseudocholinesterase)**

Sevoflurane

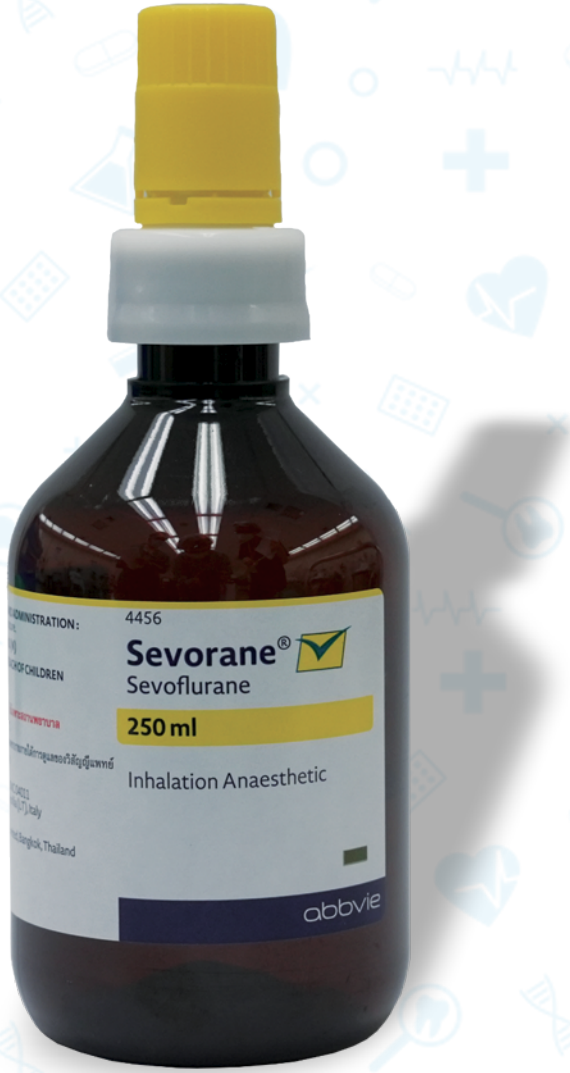

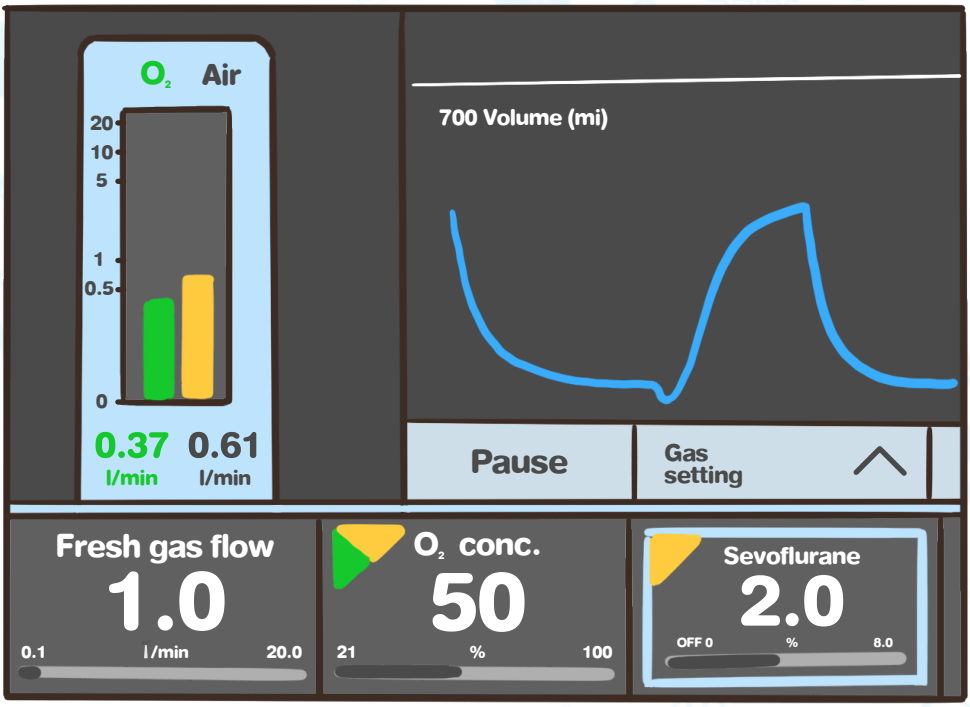

O2 Air
20
10
5
1
0.5
0
0.37 l/min
0.61 l/min
700 Volume (mi)
Pause
Gas setting
Fresh gas flow
1.0
0.1 l/min 20.0
O2 conc.
50
21 % 100
Sevoflurane
2.0
OFF 0 % 8.0

1 MAC = 2%

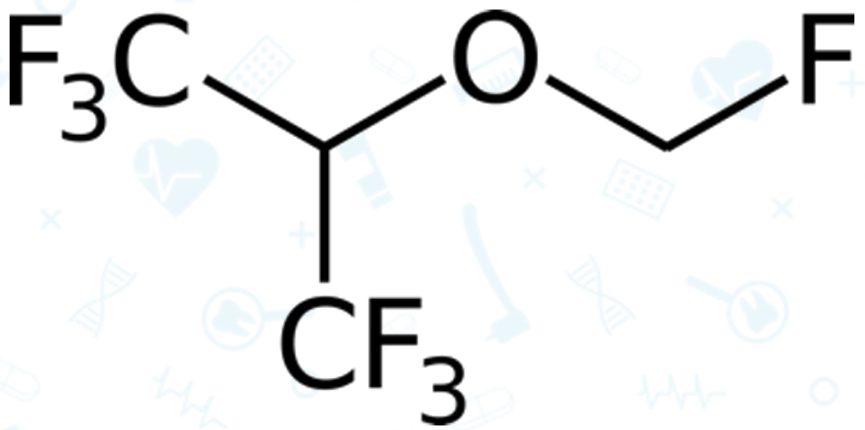

Vapor pressure at 20*C : 157 mmHg

Clinical concern

Compound A

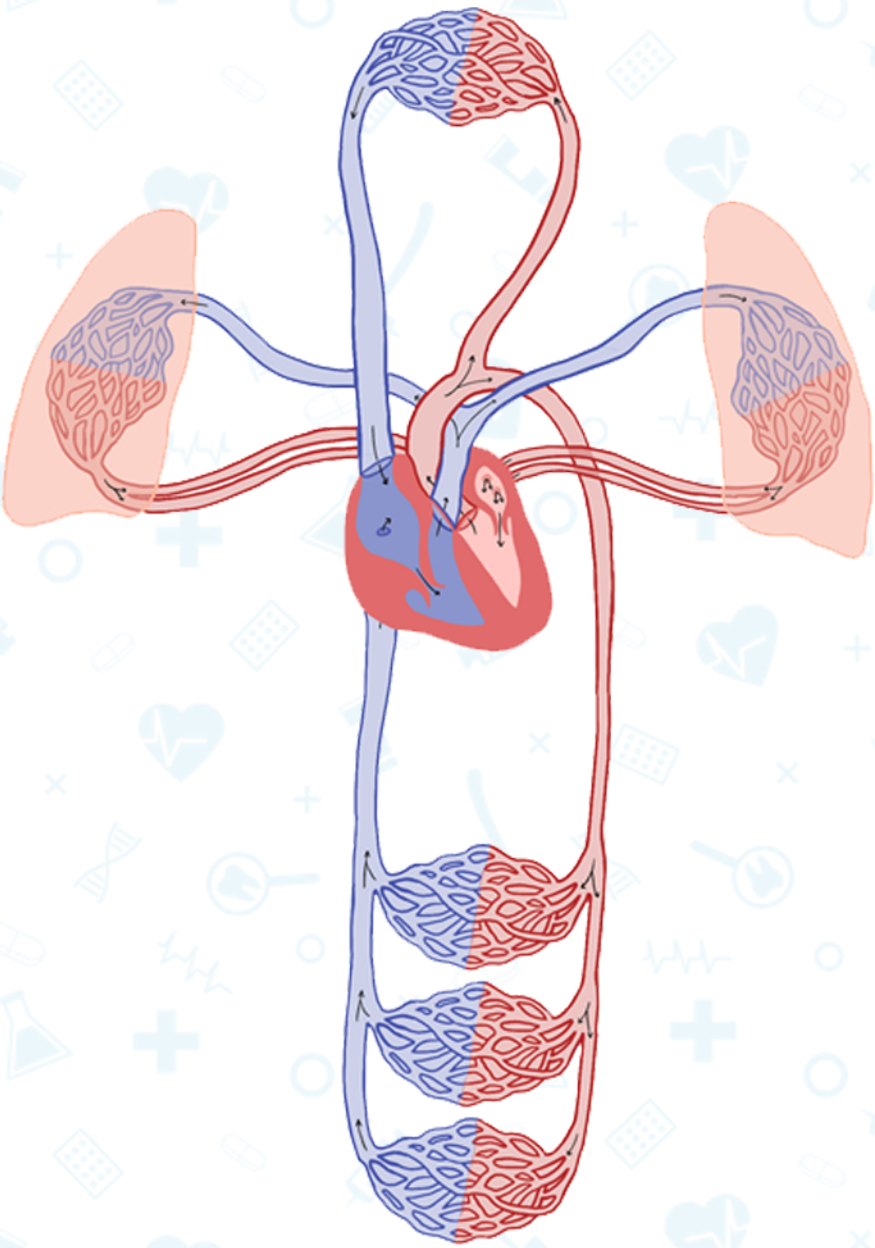

Partition coefficient

- Blood – gas : 0.65
- Muscle – blood : 3.1
- Brain – blood : 1.7
- Fat – blood : 48

Desflurane

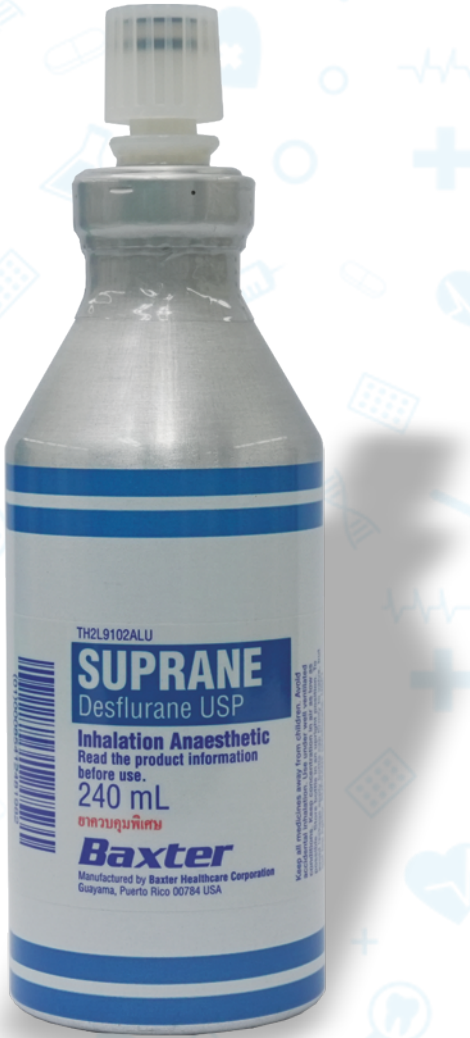
TH2L9102ALU
SUPRANE
Desflurane USP
Inhalation Anaesthetic
Read the product information before use.
240 mL
ยาควบคุมพิเศษ
Baxter
Manufactured by Baxter Healthcare Corporation
Guayama, Puerto Rico 00784 USA
Keep all medicines away from children. Avoid

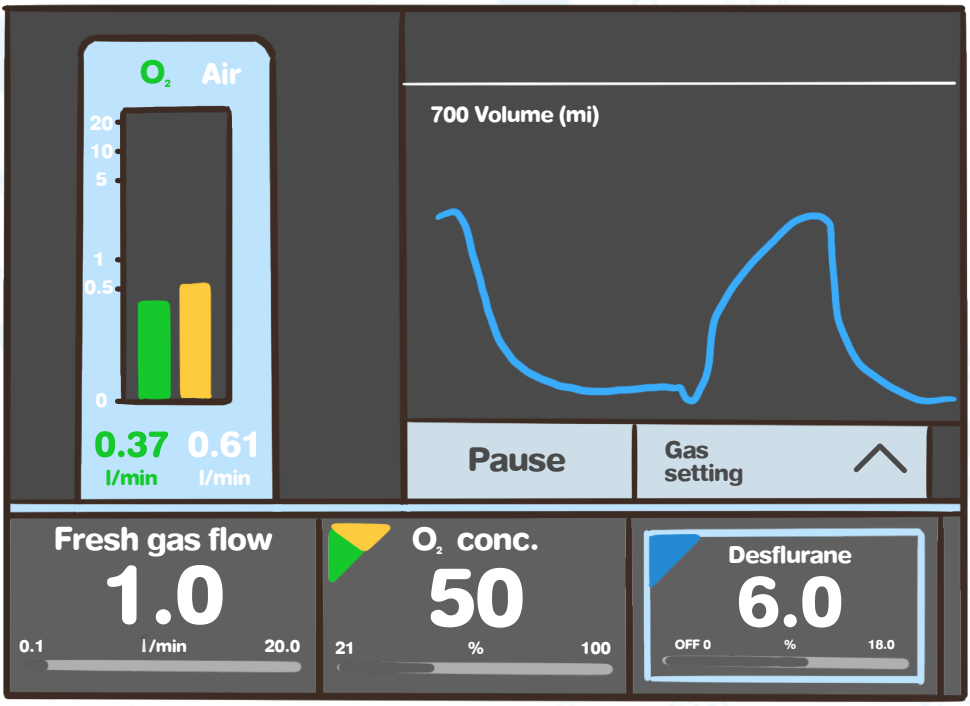

O₂ Air
20
10
5
1
0.5
0
0.37 l/min
0.61 l/min
700 Volume (mi)
Pause
Gas setting
Fresh gas flow
1.0
0.1 l/min 20.0
O₂ conc.
50
21 % 100
Desflurane
6.0
OFF 0 % 18.0

1 MAC = 6%

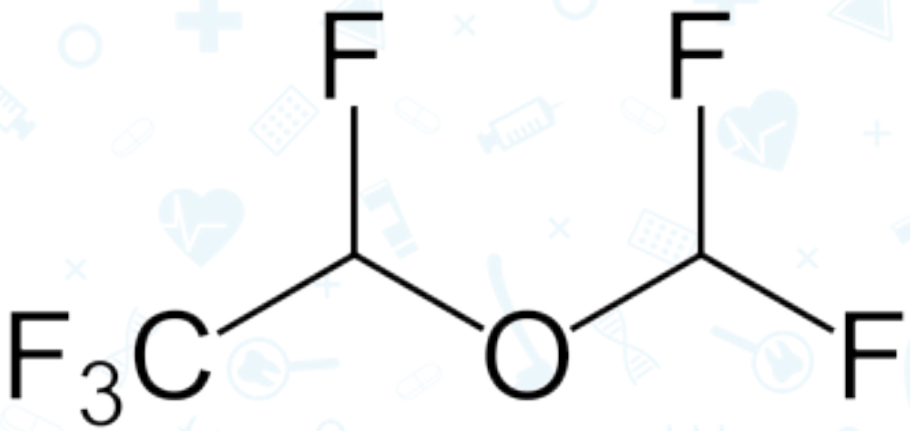

Vapor pressure at 20*C : 669 mmHg

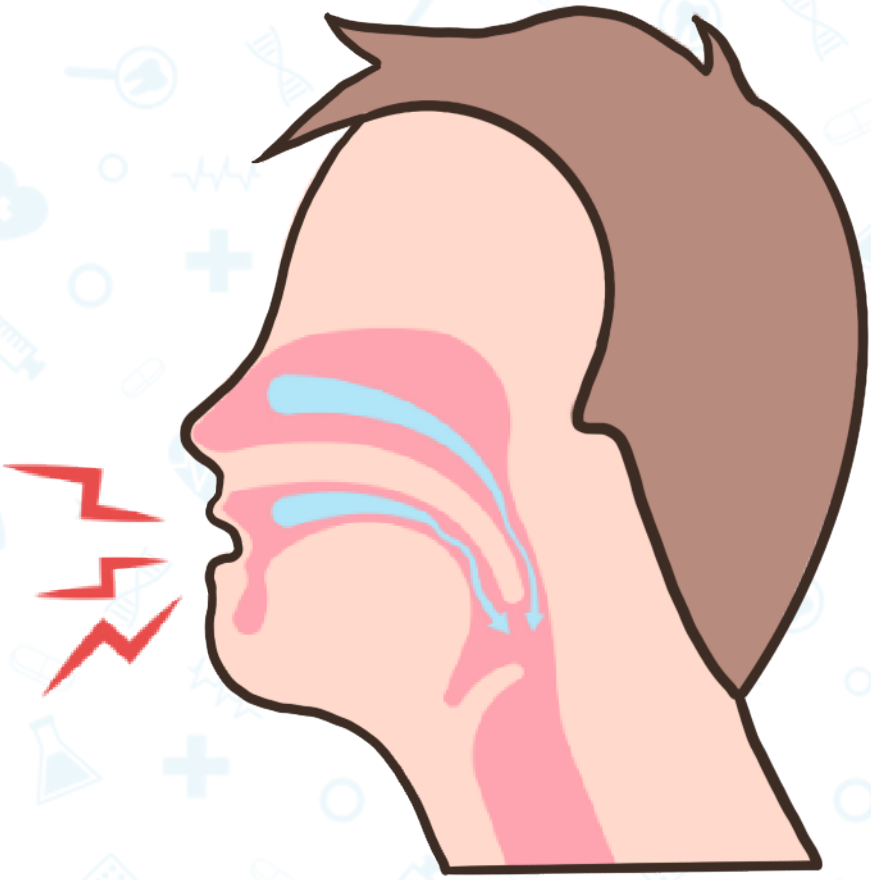

Clinical concern

Airway irritation

Partition coefficient

- Blood – gas : 0.45
- Brain – blood : 1.3
- Muscle – blood : 2.0
- Fat – blood : 27

Nitrous oxide

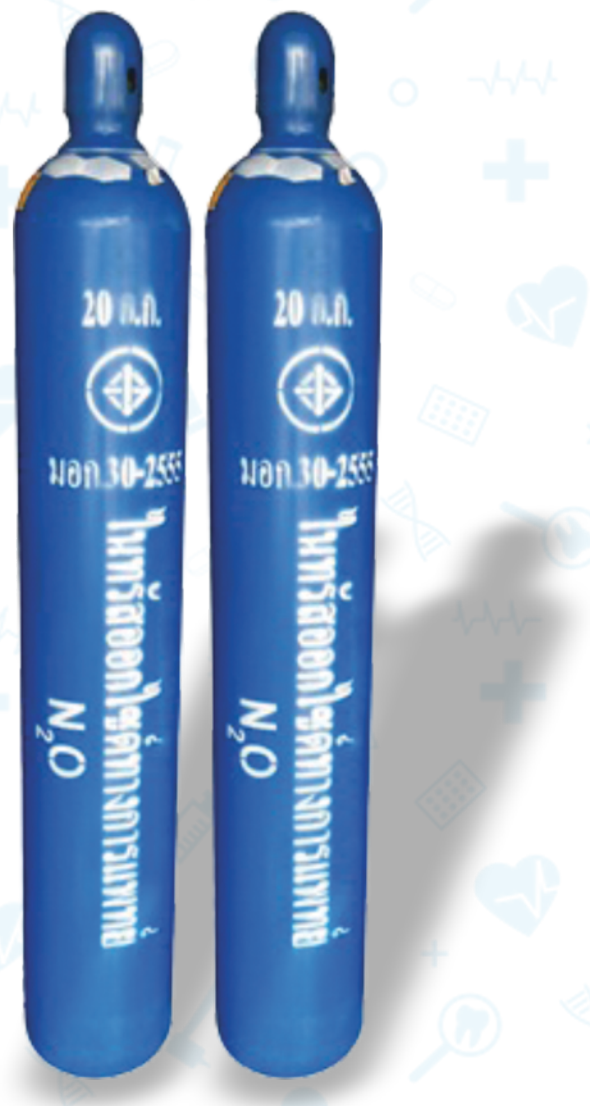

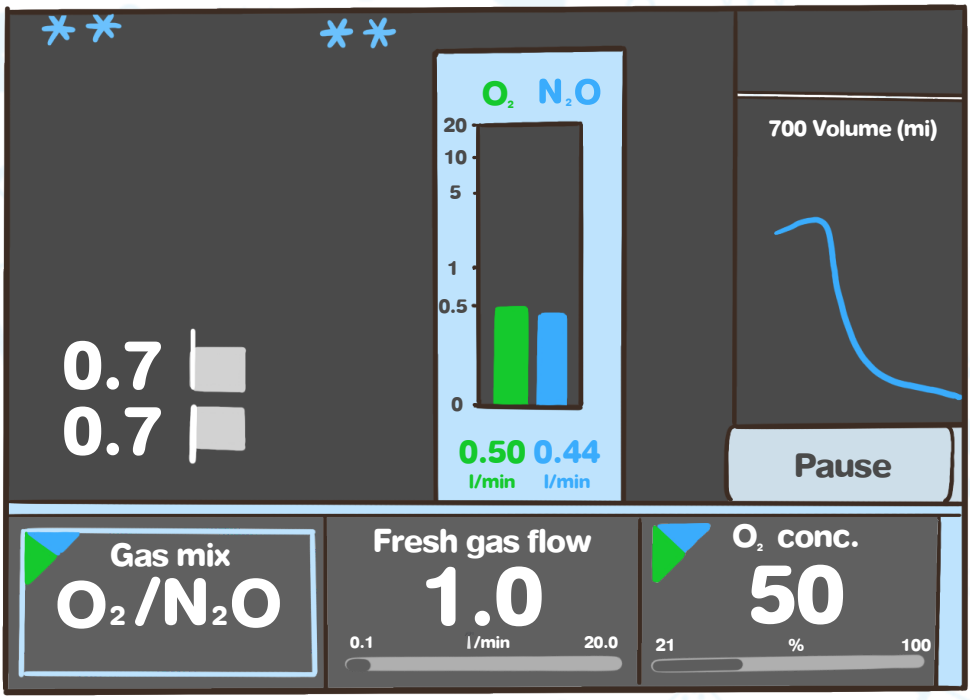

O2 N2O
20
10
5
1
0.5
0
0.50 0.44
l/min l/min
700 Volume (ml)
0.7
0.7
Pause
Gas mix
O2/N2O
Fresh gas flow
1.0
0.1 l/min 20.0
O2 conc.
50
21 % 100

1 MAC = 104%

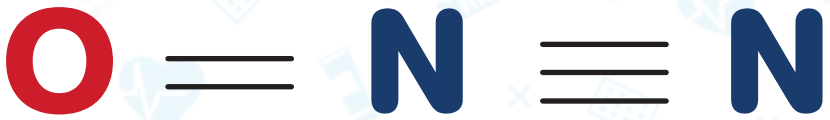

Vapor pressure at 20*C : 38, 770 mmHg

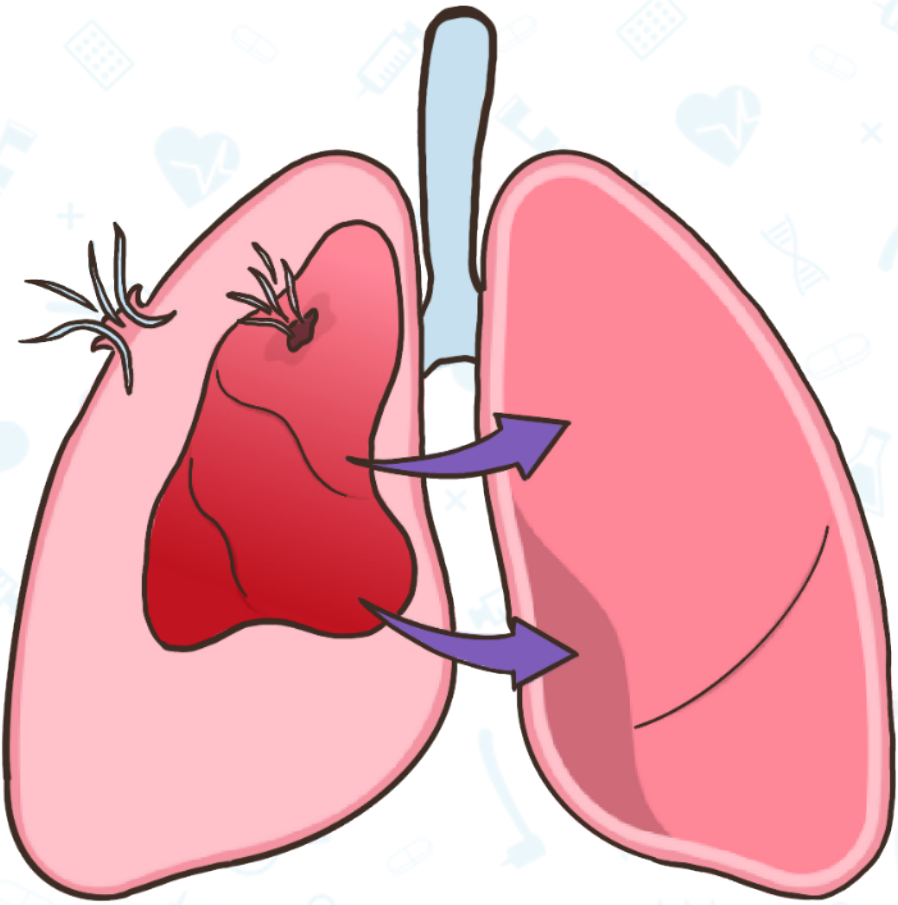

Clinical concern

Gas expansion

Contraindication

- Venous air embolism
- Pneumocephalus
- Pneumothorax
- COPD with lung bles
- Acute intestinal obstruction
- Tympanic membrane grafting
- Intraocular procedures involving gas bubbles

Partition coefficient

- Blood – gas : 0.46
- Brain – blood : 1.1
- Muscle – blood : 1.2
- Fat – blood : 2.3
