# Supplementary material for: Effectiveness of game-based learning of anesthetic drugs in anesthesiology residents: a pilot study
Source: BMC Med Educ. 2026 Jul 27;26:1700. doi: 10.1186/s12909-026-10011-5 (PMC13689327; doi:10.1186/s12909-026-10011-5)
Supplement: Supplementary file 3 [file 12909_2026_10011_MOESM3_ESM.docx]

Supplement 1 Anesthesiology Residency curriculum

**Year 1 Anesthesiology Curriculum, Learning Activities on Fridays, 2:00 PM to 4:30 PM**

First-Half Academic Schedule

| List | Topic | Activity | Date |
| --- | --- | --- | --- |
| 1 | Preoperative evaluation and preparation | Lecture | July |
| 2 | Choice of anesthesia, GA | Lecture | July |
| 3 | Intubation | Lecture | July |
| 4 | Spinal anesthesia | Lecture | July |
| 5 | Basic monitoring | Lecture | July |
| 6 | Intraoperative fluid management | Lecture | July |
| 7 | Basics in acute pain management | Lecture | July |
| 8 | Intubation and airway equipment | Workshop | July |
| 9 | Spinal block | Workshop | July |
| 10 | Respiratory anatomy and physiology | VDO lecture | July |
| 11 | Oxygen therapy | VDO lecture | July |
| 12 | Cardiovascular anatomy and physiology | VDO lecture | August |
| 13 | Central and peripheral nervous system anatomy and physiology | Topic | August |
| 14 | General concepts in pharmacology | VDO lecture | August |
| 15 | Non-opioid intravenous anesthetic agents | VDO lecture | August |
| 16 | Muscle relaxants and its antagonists | VDO lecture | August |
| 17 | Anesthetic gas and vapors | VDO lecture | August |
| 18 | Opioid | VDO lecture | August |
| 19 | Local anesthetics | VDO lecture | August |
| 20 | Autonomic drugs and central nervous system drugs | Topic | September |
| 21 | Cardiovascular drugs and diuretics | Topic | September |
| 22 | Hematologic system, antithrombotic agents, antiplatelet agents, anticoagulant agents | Topic | September |
| 23 | Blood and blood component transfusion | VDO lecture | October |
| 24 | Patient blood management | VDO lecture | October |
| 25 | Temperature regulation | Topic | October |
| 26 | Postoperative care | Topic | October |
|  | Midterm exam | November | |

Second-Half Academic Schedule

| List | Topic | Activity | Date |
| --- | --- | --- | --- |
| 28 | Epidural and caudal anesthesia | Topic | November |
| 29 | Airway management | Topic | November |
| 30 | Depth of anesthesia and awareness | Topic | December |
| 31 | Anesthesia machine and work station | VDO lecture | December |
| 32 | Vaporizer | VDO lecture | December |
| 33 | Anesthetic circuit | VDO lecture | December |
| 34 | Carbon dioxide absorption | VDO lecture | December |
| 35 | Anesthesia ventilator | VDO lecture | December |
| 36 | Scavenging system | VDO lecture | December |
| 37 | Quality and safety | Topic | December |
| 38 | Acid-base balance | Topic | January |
| 39 | Occupational hazard | Topic | January |
| 40 | Regional anesthesia | VDO lecture | January |
| 41 | Positioning and potential injuries | Topic | January |
|  | Final exam | February | |

**Year 2 Anesthesiology Curriculum, Learning Activities on Wednesdays, 2:00 PM to 4:30 PM**

| List | Topic |  |  |
| --- | --- | --- | --- |
| 1 | Anesthesia in laparoscopic surgery | Topic | August |
| 2 | Anesthesia in plastic surgery | Topic | August |
| 3 | Anesthetic consideration in herbal medicine | Topic | August |
| 4 | Anesthesia in eye surgery | Topic | September |
| 5 | Anesthesia in ENT surgery | Topic | October |
| 6 | Anesthesia in ambulatory surgery | Topic | October |
|  | Midterm exam | November | |

Second-Half Academic Schedule

| List | Topic |  |  |
| --- | --- | --- | --- |
| 7 | Acute pain management | Topic | November |
| 8 | Chronic pain management | Topic | December |
| 9 | Anesthesia in endocrine disease | Topic | December |
| 10 | Anesthesia in neuromuscular disease (i.e. myasthenia gravis) | Topic | January |
| 11 | Anesthesia in genetic disease (i.e. malignant hyperthermia) | Topic | January |
|  | Midterm exam | February | |

**Year 3 Anesthesiology Curriculum, Learning Activities on Mondays, 2:00 PM to 4:30 PM**

First-Half Academic Schedule

| List | Topic |  |  |
| --- | --- | --- | --- |
| 1 | Anesthesia in cardiac disease (ischemic heart disease, hypertension, heart failure, cardiomyopathy, cardiac tamponade, constrictive pericarditis) | Topic | August |
| 2 | Circulatory assistance and cardiopulmonary bypass | Topic | August |
| 3 | Rhythm disorder and conduction defects | Topic | September |
| 4 | Anesthesia in respiratory disease | Topic | October |
| 5 | Anesthesia in congenital heart disease | Topic | October |
| 6 | Cardiopulmonary resuscitation | ACLS and PALS | |
|  | Midterm exam | November | |

Second-Half Academic Schedule

| List | Topic |  |  |
| --- | --- | --- | --- |
| 7 | Critical care 1 (shock, sepsis, MOF, ICU score, infection control | Topic | November |
| 8 | Critical care 2 (nutrition and metabolic control) | Topic | December |
| 9 | Critical care 3 (renal replacement therapy, sedation in ICU) | Topic | December |
| 10 | Respiratory care 1 (mechanical ventilation) | Topic | December |
| 11 | Respiratory care 2 (acute respiratory failure, ARDS, non-invasive | Topic | January |
| 12 | Special technique | Topic | January |
| 13 | Anaphylaxis and anaphylactoid reaction | Topic | January |
|  | Final exam | February | |

Note: Topics are presented by residency trainees under the academic supervision and advisory of attending faculty.

Supplement 2 Pretests and posttests

1. The dose of propofol for sedation is……………………mcg/kg/min (25–75 mcg/kg/min)

2. The dose of propofol TIVA for general anesthesia is……………….mcg/kg/min

(100–200 mcg/kg/min)

3. The contraindication of thiopental is……………………………..(1 answer). (Acute intermittent porphyria)

4. The side effect of ketamine are………………………………………………….(2 answers) (increased salivation, hallucinations)

5. The reversal drug of midazolam is……………………………………..(Flumazenil)

6. The induction dose of etomidate is………………………………..mg/kg (0.2–0.3 mg/kg)

7. The side effects of etomidate are…………………………………………(2 answers)

(Involuntary myoclonic movements and transient adrenal suppression)

8. What drug is not the proper induction drug for the patient who is planned for exploratory laparotomy due to gut obstruction, dehydration, decreased urine output? The patient’s blood pressure was 85/40 mmHg, and PR was 130/min. …………………..(1 answer). (Propofol)

9. The blood-gas partition coefficient of sevoflurane is…………………(0.65)

10. The contraindication of nitrous oxide are………………………………….(2 answers) (Venous air embolism, Pneumocephalus, Pneumothorax, COPD with lung blebs, Acute intestinal obstruction, Tympanic membrane grafting, Intraocular procedures involving gas bubbles)

11. Name a drug in the aminosteroid non-depolarizing muscle relaxant group?................... (1 answer). (Cisatracurium)

12. The intubating dose of succinylcholine ……………………mg/kg (1 answer). (1–1.5 mg/kg)

13. The duration of onset of cisatracurium is ………………..min (1 answer). (3–5 mins)

14. What muscle relaxant drugs can be selected for a patient with a history of being electrocuted and sustaining burn injuries 12 hours prior to hospital arrival, who needs to undergo surgery for irrigation and debridement? ............... (2 answer)

(Succinylcholine, Rocuronium, Cisatracurium).

15. What is the intubating dose of rocuronium for a patient at risk of aspiration due to a full stomach? ................ mg/kg (answer with 1 option) (1–1.2 mg/kg)

16. What are the contraindications for the use of succinylcholine? ............................................ (2 answer) (Hyperkalemia, Increase ICP, Increase intraocular pressure)

17. Sugammadex is the antidote for which drug? ................... (1 answer). (Rocuronium)

18. What is the elimination process of cisatracurium? ................... (1 answer). (Hofmann elimination, Ester hydrolysis)

19. What is the value of 1 MAC for desflurane? ................. (1 answer). (6%)

20. What are the precautions for using desflurane? ....................... (1 answer). (Airway irritation)

Supplement 3: Baseline characteristics of the study participants

| Variable | n = 19 |
| --- | --- |
| Sex |  |
| Female | 11 (57.9%) |
| Male | 8 (42.1%) |
| Class |  |
| First-year resident | 8 (42.1%) |
| Second-year resident | 4 (21.1%) |
| Third-year resident | 7 (36.8%) |

Supplement 4: Comparison of knowledge between pre- and posttests, classified by each year of residence

| Variables | Pretest | Posttest | p-value |
| --- | --- | --- | --- |
| First-year resident |  | | |
| Induction drug (8) | 4.63 (3.94, 5.50) | 7.00 (6.94, 7.44) | 0.022 |
| Volatile anesthetics (4) | 2.75 (2.00, 3.00) | 4.00 (4.00, 4.00) | 0.021 |
| Neuromuscular agent (8) | 7.00 (6.88, 7.63) | 7.75 (6.875, 8.00) | 0.168 |
| Total score (20) | 13.88 (13.31, 14.88) | 18.62 (17.94, 19.25) | 0.014 |
| Second-year resident |  | | |
| Induction drug (8) | 5.13 (4.13, 5.81) | 7.63 (7.25, 8.00) | 0.125 |
| Volatile anesthetics (4) | 2.75 (2.50, 3.00) | 4.00 (3.75, 4.00) | 0.174 |
| Neuromuscular agent (8) | 6.25 (5.75, 6.63) | 7.63 (7.50, 7.81) | 0.125 |
| Total score (20) | 13.25 (12.88, 14.06) | 19.38 (18.94, 19.50) | 0.125 |
| Third-year resident |  | | |
| Induction drug (8) | 5.50 (5.13, 5.75) | 8.00 (7.75, 8.00) | 0.022 |
| Volatile anesthetics (4) | 2.75 (2.25, 3.00) | 4.00 (4.00, 4.00) | 0.021 |
| Neuromuscular agent (8) | 6.50 (6.13, 6.75) | 7.50 (7.13, 8.00) | 0.016 |
| Total score (20) | 14.75 (13.25, 15.25) | 19.25 (19.00, 20.00) | 0.016 |
